# Structural Dependence of Extended Amide III Vibrations in Two-Dimensional Infrared Spectra

**DOI:** 10.1021/acs.jpclett.3c02662

**Published:** 2023-10-09

**Authors:** Julia Brüggemann, Maria Chekmeneva, Mario Wolter, Christoph R. Jacob

**Affiliations:** Technische Universität Braunschweig, Institute of Physical and Theoretical Chemistry, Gaußstraße 17, 38106 Braunschweig, Germany

## Abstract

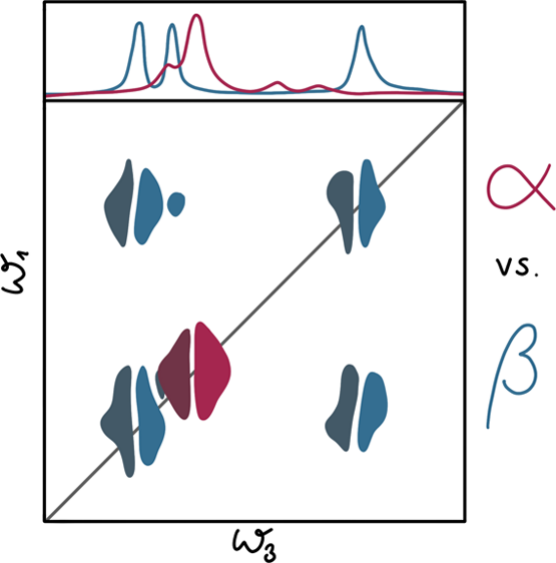

Two-dimensional infrared (2D-IR) spectroscopy is a powerful experimental method for probing the structure and dynamics of proteins in aqueous solution. So far, most experimental studies have focused on the amide I vibrations, for which empirical vibrational exciton models provide a means of interpreting such experiments. However, such models are largely lacking for other regions of the vibrational spectrum. To close this gap, we employ an efficient quantum-chemical methodology for the calculation of 2D-IR spectra, which is based on anharmonic theoretical vibrational spectroscopy with localized modes. We apply this approach to explore the potential of 2D-IR spectroscopy in the extended amide III region. Using calculations for a dipeptide model as well as alanine polypeptides, we show that distinct 2D-IR cross-peaks in the extended amide III region can potentially be used to distinguish α-helix and β-strand structures. We propose that the extended amide III region could be a promising target for future 2D-IR experiments.

Vibrational spectroscopy allows one to directly probe the structure and dynamics of biomolecules in aqueous solution.^1−6^ Two-dimensional infrared (2D-IR) spectroscopy^7−11^ has been shown to sensitively probe the secondary and tertiary structure of biomolecules^12−18^ and can be used to elucidate the ultrafast dynamics of protein folding.^19−24^ By spreading out the spectroscopic information over two dimensions, 2D-IR provides additional information about anharmonicities and couplings between vibrational modes. Thus, it provides more detailed information than conventional one-dimensional infrared spectroscopy.

2D-IR spectroscopy for proteins is predominantly done within the amide I region^25−33^ (i.e., probing the coupled carbonyl stretching vibrations of the amide groups). Besides experimental considerations—most importantly the fact that the amide I vibrations show a particularly strong infrared intensity—this is facilitated by the availability of empirical exciton models^34^ for the amide I vibrations. Such models can be used for efficiently predicting 2D-IR spectra in the amide I region.^35−41^ Conversely, the lack of such models beyond the amide I vibrations—and thus the inability of making reliable computational predictions—precludes the interpretation of 2D-IR spectra in other spectral regions and hampers the application of 2D-IR spectroscopy beyond the amide I vibrations. Exceptions include the use of artificial amino acids or of specific functional groups as labels for 2D-IR spectroscopy.^23,24,42−45^

So far, only very few studies have explored 2D-IR spectroscopy using protein backbone vibrations beyond the amide I region. DeFlores et al. as well as Maekawa et al. investigated the cross peaks between the amide I and the amide II vibrations^46−51^ and demonstrated that these are highly sensitive to secondary structure. Despite some effort for parametrizing suitable exciton models,^52,53^ the interpretation of such experiments remains partly elusive.

Here, we propose that the extended amide III region (between ca. 1200 and 1400 cm^–1^)^54−58^ should be another promising target for 2D-IR spectroscopy, and predict that the cross-peaks in this region will be highly sensitive to the secondary structure. This suggestion is backed up by our recently developed methodology for the efficient quantum-chemical calculation of 2D-IR spectra,^59^ which allows for the prediction of 2D-IR spectra for arbitrary molecular systems in any spectral region, without the need for an empirical parametrization.

In the extended amide III region, one finds the “classical” amide III vibration of the peptide groups, which is an in-phase combination of the N–H in-plane bending and the C–N stretching vibration as well as two C^α^–H bending vibrations. However, these three vibrations are strongly coupled,^54−56^ and furthermore couple with the corresponding vibrations of other residues, resulting in delocalized extended amide III vibrations. Previously, it was shown^60^ that by performing a localization of normal modes^61^ in the extended amide III region, a local-mode picture can be recovered, in which the local-mode frequencies as well as their harmonic coupling constants can be directly related to the backbone dihedral angles. While this structural dependence is difficult to exploit for extracting structural information from conventional, one-dimensional vibrational spectra,^62^ 2D-IR spectra sensitively depend on these vibrational couplings between local modes.

As a first model system, we consider the dipeptide *N*-acetyl-l-alanine-*N*-methylamide, which was previously studied in ref (60). Here, we find four normal modes in the extended amide III region (see Figure 1a as well as Figures S1 and S2 in the Supporting Information). Two of these (normal modes 1 and 2) are dominated by the C^α^–H bending vibrations at the central C^α^ atom, whereas the other two (normal modes 3 and 4) are dominated by the classical amide III vibrations of the two peptide groups. However, for all four normal modes, the contributions of the C^α^–H bending vibrations and the two classical amide III vibrations mix rather strongly.

**Figure 1:**
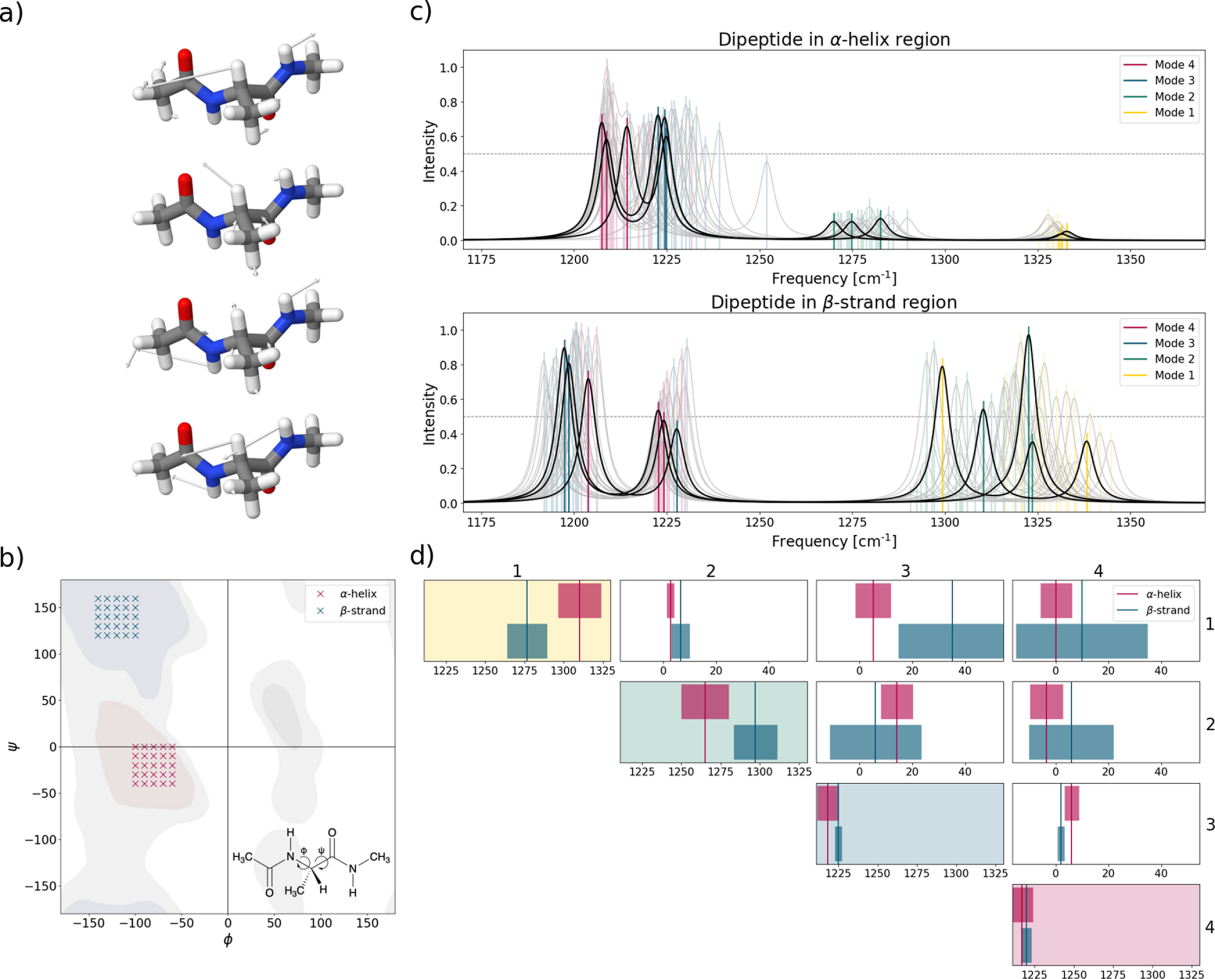
(a) Normal modes of the dipeptide *N*-acetyl-l-alanine-*N*-methylamide (with ϕ = −120° and ψ = 140°). (b) Locations of considered structures on the Ramachandran plot (adapted from ref (63)). (c) Calculated one-dimensional infrared spectra (L-VSCF/L-VCI) for α-helix conformations (upper part) and β-strand conformations (lower part). The solid black lines refer to the three structures also considered in Figure 2 below, whereas all 25 considered structures are included as gray lines. Additional plots showing a comparison of the 1D spectra are shown in Figures S3–S6. The assignment of the transitions is based on the harmonic normal modes. (d) Comparison of mean coupling matrices (local-mode frequencies on the diagonal and harmonic couplings in the off-diagonal) and standard deviations (all values in cm^–1^) over all structures considered in the α-helix and β-strand regions.

For this model dipeptide, we consider 25 structures (with ϕ = −100°, ..., −60° and ψ = −40°, ..., 0°) in the α-helix region of the Ramachandran plot and 25 structures (with ϕ = −140°, ..., −100° and ψ = +120°, ..., +160°) in the β-strand region (see Figure 1b). For these sets of structures, we performed harmonic calculations of the infrared spectra, from which we extracted the four relevant normal modes. Next, we localized the normal modes,^61^ which results in localized modes that describe the two local amide III vibrations (localized modes 3 and 4) and the two pure C^α^–H bending vibrations^60^ (localized modes 1 and 2). These localized modes served as basis for calculations of the anharmonic one- and two-dimensional infrared spectra with localized-mode vibrational self-consistent field (L-VSCF) and localized-mode vibrational configuration interaction (L-VCI),^64,65^ using anharmonic one-mode potentials and harmonic two-mode potentials. Further details are given in the Computational Details below and in the Supporting Information (see section S1).

Figure 1c shows an overlay of the anharmonic 1D infrared spectra of the dipeptide systems in the α-helix and β-strand regions. The mean values of the frequencies of the four modes as well as the standard deviations over the 25 structures considered in each case are listed in the Supporting Information (Table S7 and Figure S7). Overall, the plots show that the vibrational frequencies and intensity patterns strongly depend on the secondary structure. Normal mode 4 (dominated by the in-phase combination of the two classical amide III vibrations) in the α-helix region is shifted by about 10 cm^–1^ to lower wavenumbers than the corresponding peaks in the β-strand region. The positions of normal mode 3 (dominated by the out-of-phase combination of the two classical amide III vibrations) differ largely, with the peaks in the α-helix region shifted by about 23 cm^–1^ to higher wavenumbers compared to those in the β-strand region. For both normal modes, similar standard deviations are found. For normal mode 2 (dominated by C^α^–H bending), the peaks for the structures in the α-helix region are downshifted by about 28 cm^–1^ compared to those for the structures in the β-strand region, and their standard deviation in the β-strand region is almost twice as large as in the α-helix region. Finally, for normal mode 1 (also dominated by C^α^–H bending), the peaks appear at about the same frequency for both regions, but they differ substantially in their standard deviations. For the α-helix region, the peak position shows the lowest standard deviation overall, whereas for the β-strand region, the peaks are broadly scattered. While for the structures in the α-helix region the intensities of the C^α^–H bending vibrations (normal modes 1 and 2) are comparably small, they are significantly larger for those in the β-strand region, but in the latter case, the intensities also vary widely between different structures.

The one-dimensional infrared spectra clearly demonstrate the large structural sensitivity of the vibrations in the extended amide III region (see also ref (60)), but the large variations of peak positions and intensities within the two considered structural motifs complicate a use for a direct structural assignment. However, when switching to a local-mode picture, the relationship between local-mode frequencies, harmonic coupling constants, and the backbone dihedral angles becomes more direct. For a detailed discussion, we refer to ref (60). Figure 1d visualizes the mean values and standard deviations of the local-mode frequencies and the harmonic coupling constants for the structures considered in the α-helix and in the β-strand region (see Tables S8 and S9 in the Supporting Information for underlying data). Here, clear distinctions (with nonoverlapping standard deviations) can be found between the two structural motifs in the coupling constants. For instance, the coupling between the two classical amide III vibrations (localized modes 3 and 4) is rather small for the β-strand region, and significantly larger for the α-helix region. Conversely, the coupling between localized mode 1 (C^α^–H bending) and localized mode 3 (classical amide III) is substantially larger for the β-strand region compared to that of the α-helix region.

These differences in the coupling constants are explicitly revealed in the 2D-IR spectra. Calculated 2D-IR spectra for three exemplary structures from the α-helix and β-strand regions, respectively, are shown in Figure 2a,b. The full sets of calculated spectra for all considered structures are shown in the Supporting Information (see Figures S8 and S9). For the two different structural motifs, the calculated 2D-IR spectra show very distinctive patterns, with the diagonals reflecting the 1D spectra discussed above. For the structures from the α-helix region, the two classical amide III vibrations (normal modes 3 and 4) show the strongest diagonal peaks, with an intense cross-peak between them. For the structures from the β-strand region, this cross-peak is absent. However, the diagonal peaks corresponding to the C^α^–H bending vibrations (normal modes 1 and 2) are now clearly visible, and there are strong cross peaks between those and the classical amide III vibrations, i.e., between normal modes 3 or 4 and normal mode 1. These cross peaks could possibly be used as a signature of the specific configuration of backbone angles that are characteristic for the two considered structural motifs.

**Figure 2:**
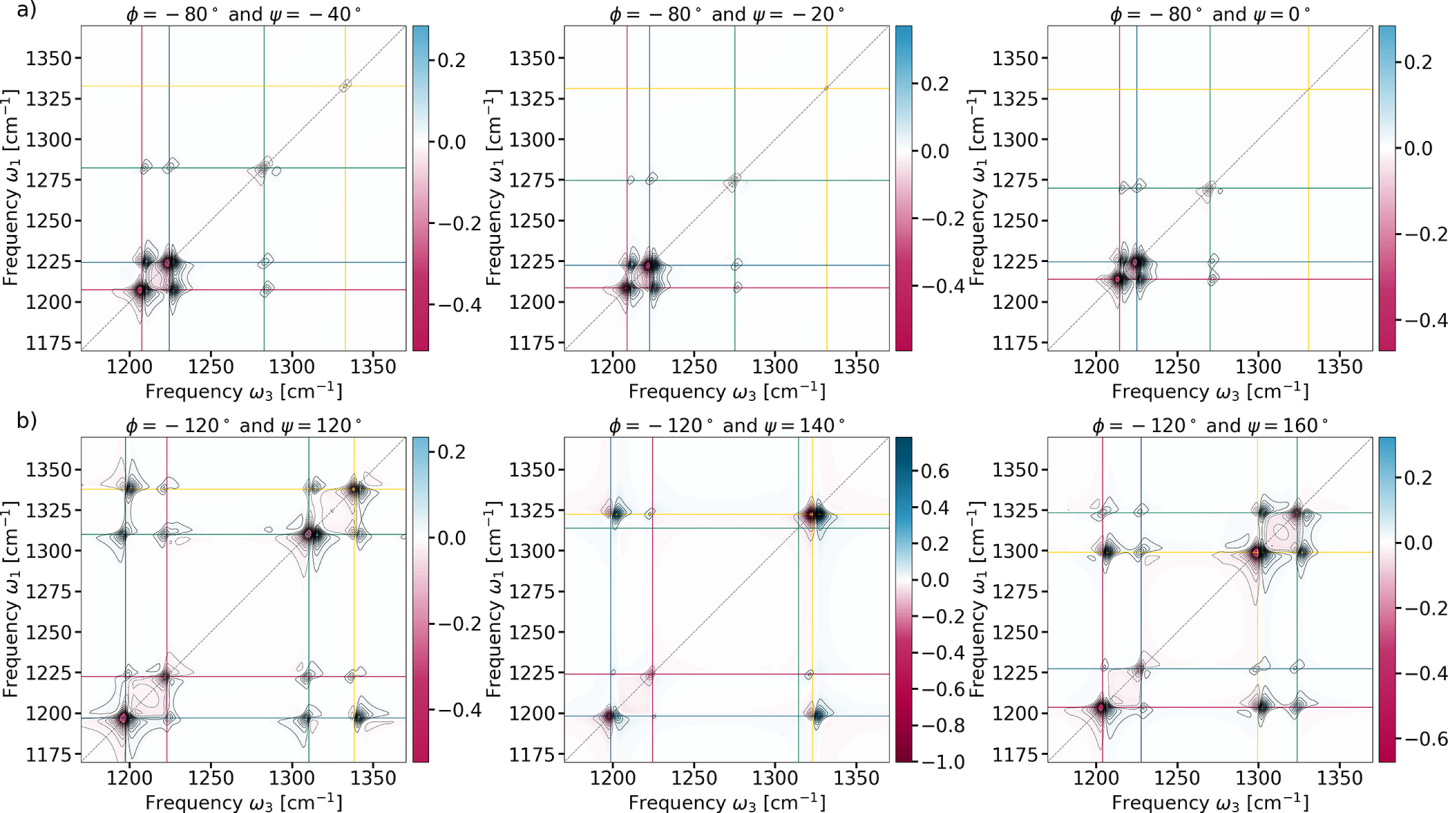
Calculated 2D-IR spectra (L-VSCF/L-VCI) of the *N*-acetyl-l-alanine-*N*-methylamide dipeptide (a) in α-helix conformation with ϕ_α_ = −80° and ψ_α_ = −40°, – 20°, 0° and (b) β-strand conformation with ϕ_β_ = −120° and ψ_β_ = 120°, 140°, 160°.

To assess to what extent these signatures are preserved for larger systems, we first consider two polypeptides consisting of ten alanine residues (with the termini capped with methyl groups) with an idealized α-helical structure (ϕ_α_ = −60°, ψ_α_ = −40°) and an idealized β-strand structure (ϕ_β_ = −135°, ψ_β_ = 135°). In addition, as a model of a β-sheet we consider a system consisting of two antiparallel polyalanine-5 β-strands (with ϕ = −138.6°, ψ = 134.5°, and ω = 178.5°, adapted from ref (66)). The 2D-IR spectra calculated for these model polypeptides are shown in Figure 3a. We find that in all three cases, the classical amide III vibrations lie up to a wavenumber of around 1250 cm^–1^, whereas the C^α^–H bending vibrations are found between ca. 1260 and 1340 cm^–1^, in qualitative agreement with the results for the dipeptide model.

**Figure 3:**
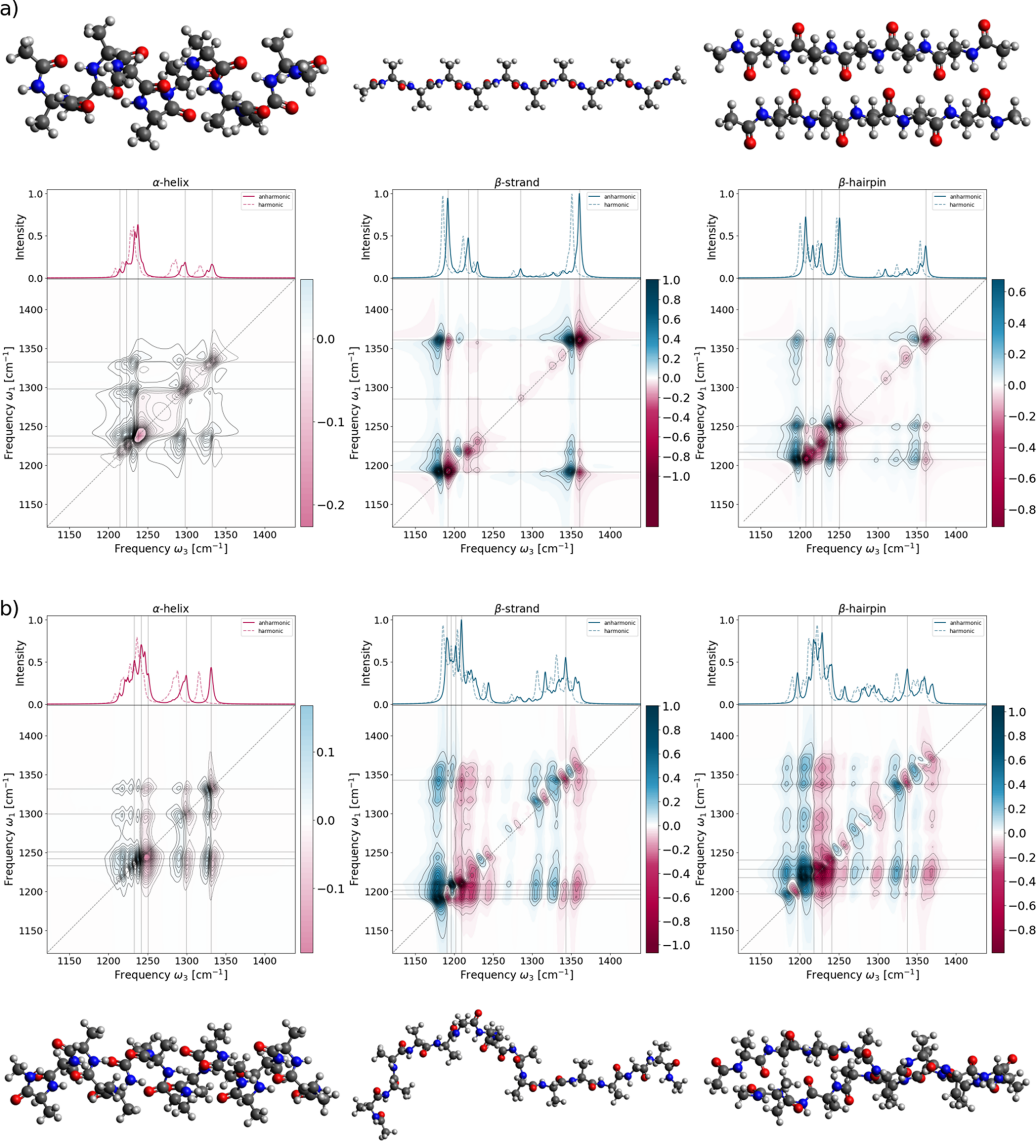
Calculated 2D-IR spectra (L-VSCF/L-VCI) (a) of polyalanine-10 in an idealized α-helical conformation, of an idealized β-strand conformation, and of two antiparallel polyalanine-5 β-strands and (b) of a snapshot of polyalanine-15/16 extracted from a molecular dynamics simulation of an α-helix, a β-strand, and a β-hairpin. In the insets above the 2D-IR spectra, the calculated harmonic and anharmonic infrared spectra are shown for comparison. Horizontal and vertical lines indicate the five largest peaks of the 1D spectra. The molecular structures are shown above and below the corresponding spectra.

The 2D-IR spectrum for the α-helical polyalanine-10 has a strikingly low intensity compared to that for the β-strand structure, with the classical amide III band as its most intense peak. As there is only one strong peak for the classical amide III vibration, cross-peaks between the amide III vibrations are hardly visible. Similarly, there are only very weak peaks for the C^α^–H bending vibrations, and cross-peaks between the classical amide III and C^α^–H bending vibrations are similarly weak. For the β-strand, both the classical amide III vibrations and the C^α^–H bending show strong signals on the diagonal. Here, the cross-peaks between the classical amide III vibrations are rather weak, while there are strong cross-peaks between the classical amide III and the C^α^–H bending vibrations. This pattern is preserved for the β-sheet model (consisting of two antiparallel β-strands), even though the cross-peaks between the classical amide III vibrations gain some intensity. Overall, this is in qualitative agreement with the dipeptide model and indicates that the identified signatures for distinguishing α-helical and β-strand structures could be transferable to larger systems.

In addition, we considered three polypeptides consisting of 15 or 16 alanine residues, with structures that were extracted from a molecular dynamics simulation (see section S1.1 in the Supporting Information for details). These represent polyalanine-15 in an α-helical and in a β-strand conformation, as well as a polyalanine-16 in the conformation of a β-hairpin (the additional alanine residue is used to connect the two antiparallel β-strands). However, as all three structures of polyalanine-15/16 are not stable during the molecular dynamics simulation, they start to deviate substantially from idealized structures (see Figure 3b), and some of their dihedral angles do not fit into their respective regions on the Ramachandran plot anymore (see Figures S10–S12 in the Supporting Information). While the α-helix and the β-hairpin are still recognizable, the β-strand structure starts to approach a random coil. Results for additional snapshots along the molecular dynamics trajectory are shown in Figures S15–S17 in the Supporting Information.

The calculated 2D-IR spectra for these structures of polyalanine-15/16 are shown in Figure 3b. These larger and more distorted structures feature considerably more peaks in the 1D infrared spectra, leading to overall more complex 2D-IR spectra. Nevertheless, these largely follow the pattern already observed for the idealized structures. For the α-helical polyalanine-15, the 2D-IR spectrum has a rather low intensity, with the classical amide III vibration giving rise to the strongest peak. The C^α^–H bending vibrations as well as their cross-peaks with the classical amide III vibrations gain some intensity compared to the idealized structures but remain rather weak. In contrast, for the β-strand and the β-hairpin, the C^α^–H bending vibrations show a strong peak on the diagonal as well as visible cross-peaks with the classical amide III vibrations. Compared to the idealized structures, these are somewhat weaker but can still serve to clearly distinguish the α-helical and β-strand structures.

Overall, our computational results show that the extended amide III region is a promising target for extracting structural information from the 2D-IR spectra. In particular, it can be expected that the cross-peaks between the classical amide III vibrations and the C^α^–H bending vibration show distinct differences for different structural motifs. Therefore, we propose to investigate the extended amide III region more closely experimentally. While this might be complicated by overlap of the extended amide III vibrations with solvent background, we hope that this work makes a compelling case for overcoming such experimental challenges. We are confident that further combined experimental and computational studies will be able to establish signatures of secondary structure elements in amide III 2D-IR spectroscopy. Beyond the static structures considered here, future computational studies will have to account for structural fluctuations and solvent effects, and work in this direction is currently ongoing in our group.

More generally, we believe that our work opens up new perspectives for future 2D-IR experiments that go beyond the molecular systems and vibrational regions studied so far. By enabling the efficient quantum-chemical calculation of 2D-IR spectra,^59^ it becomes possible to suggest promising 2D-IR experiments and to lay the theoretical foundations for their interpretation. We expect that this possibility will facilitate future 2D-IR experiments that would otherwise not be attempted because of the lack of computational tools for their interpretation. The study presented here is only a first example of such possible novel directions in 2D-IR spectroscopy.

## Computational Details

The geometries of all initial molecular structures were optimized using density-functional theory (DFT) with the Turbomole V7.6 program package^67^ (Turbomole V7.1 for the polyalanine-15 structures) employing the BP86 exchange-correlation functional^68,69^ and the def2-TZVP basis set.^70^ Harmonic vibrational frequencies and normal modes were then calculated using the SNF program from the MoViPac suite.^71,72^ Both steps were automated using our PyADF scripting framework.^73,74^ All vibrational spectra have been calculated for the nondeuterated structures.

For the calculation of 2D-IR spectra, we employed the methodology previously established in ref (59). Briefly, the extended amide III modes were localized using our LocVib Python package,^61,72,75^ resulting in localized-mode coordinates, localized-mode harmonic frequencies, transition dipole moments, and harmonic coupling constants.^61^ Within these localized-mode coordinates, anharmonic one-mode potentials were calculated using Turbomole/BP86/def2-TZVP, and anharmonic frequencies and transition dipole moments were calculated with L-VSCF/L-VCI including single and double excitations (L-VCISD) using our Vibrations code.^64,65,76^

These anharmonic frequencies and transition dipole moments were used as input for the calculation of 2D-IR spectra as described in ref (59). The dephasing time was set to *T*_2_ = 1 ps, the time delay between the laser pulses was set to *t*_2_ = 0 ps, and the polarization condition was set to ⟨*ZZZZ*⟩. Results for ⟨*ZZXX*⟩ and ⟨*ZXXZ*⟩ polarization conditions are shown in Figures S13 and S14. Further details on the computational methodology are provided in section S1 of the Supporting Information. All plots of spectra were prepared using Matplotlib.^77,78^

## Data Availability

The data underlying this study are available within this article and its Supporting Information. Coordinates of all optimized molecular structures are available in Zenodo at DOI: 10.5281/zenodo.8406331.

## Supporting Information Available

Additional computational details and description of the computational methodology, additional calculated one-dimensional infrared and 2D-IR spectra for all 50 considered dipeptides, additional calculated 2D-IR spectra for snapshots of polyalanine-15/16 (PDF)
