# Supplementary material for: Structural Dependence of Extended Amide III Vibrations in Two-Dimensional Infrared Spectra
Source: J Phys Chem Lett. 2023 Oct 9;14(41):9257–64. doi: 10.1021/acs.jpclett.3c02662 (PMC10591501; doi:10.1021/acs.jpclett.3c02662)
Supplement: Supplementary file 1 — jz3c02662_si_001.pdf [file jz3c02662_si_001.pdf]

Supporting Information: Structural Dependence of Extended Amide III Vibrations in Two-Dimensional Infrared Spectra

Julia Brüggemann, Maria Chekmeneva, Mario Wolter, and Christoph R. Jacob*

Technische Universität Braunschweig, Institute of Physical and Theoretical Chemistry, Gaußstraße 17, 38106 Braunschweig, Germany

E-mail: c.jacob@tu-braunschweig.de

Contents

S1 Computational Methodology **S3**

S1.1 Molecular structures S3

S1.2 Localization of normal modes S4

S1.3 L-VSCF/L-VCI S4

S1.4 Calculation of 1D and 2D-IR spectra S6

S2 Additional data for the dipeptide molecule **S7**

S2.1 Additional information for the dipeptide in the α-helix region with the angles $\phi_\alpha = -80$, $\psi_\alpha = -20$ S7

S2.2 Additional information for the dipeptide in the β-strand region with the angles $\phi_\beta = -120$, $\psi_\beta = 140$ S9

S2.3 1D harmonic and anharmonic spectra S11

S2.4 Mean values of frequencies and coupling matrices for the dipeptide S15

S2.5 Overview of all 2D spectra and their anharmonicities of fundamental modes . S16

S3 Additional data for the polyalanine molecules **S18**

References **S35**

S1 Computational Methodology

S1.1 Molecular structures

For the dipeptide N-acetyl-L-alanine-N-methylamide, molecular structures with the chosen backbone dihedral angles were constructed. Subsequently, these structures were each optimized while keeping the dihedral angles ϕ and ψ fixed at the chosen values.

The polyalanine-10 models consist of ten alanine units, where the terminal carboxylic acid and amine groups are capped by a methyl group. Here, idealized structures were generated using AVOGADRO V1.2.0.[1,2] For the α-helical structure, we used $\phi_\alpha = -60°, \psi_\alpha = -40°$, whereas for the β-strand structure, we used $\phi_\beta = -135°, \psi_\beta = 135°$. For the β-sheet structures, idealized backbone angles of $\phi_{sheet} = -138.6°, \psi_{sheet} = 134.5°$ and $\omega_{sheet} = 178.5°$ were adapted from the crystalline structure of the antiparallel poly-L-alanine β-sheet[3] for two strands placed in vicinity of each other. These idealized structures were then optimized with fixed backbone dihedral angles. The lengths of the hydrogen bonds between the two strands of the idealized β-sheet are between 192.2 and 208.1 pm.

To construct the polyalanine-15/16 models, we performed molecular dynamics simulations, starting at idealized α-helix, β-strand, and β-hairpin structures, respectively. Again, the termini are capped by methyl groups. These initial structures were generated using the molfacture function featured in VMD.[4] The topology was created using the AMBER99SB-ILDN force field.[5] The molecular dynamics (MD) simulations were done with GROMACS,[6] using the water model TIP3P[7] and standard equilibration methods at room temperature and normal pressure. As the MD simulations show, both systems start do deform. We took a snapshot from the early phase of each of the MD simulations. Results for additional snapshots from later points in the same trajectories are shown in Figs. S15–S17 below. All these snapshots were then optimized quantum-chemically with fixed backbone-dihedral angles.

Coordinates of all optimized molecular structures are provided in the data set at Ref. 8.

S1.2 Localization of normal modes

After performing a harmonic frequency calculation, we identify the modes belonging to the extended amide III region. For the dipeptides, these can be identified straightforwardly based on their wavenumbers. For the polyalanine-10 and polyalanine-15 models, the assignment used here is listed in Tables S10 to S18.

For the subset of these extended amide III modes, a unitary transformation $\boldsymbol{U}$ is performed, such that the resulting modes,

$$\tilde{\boldsymbol{Q}}^{sub} = \boldsymbol{Q}^{sub}\boldsymbol{U} \tag{S1}$$

are maximally localized.[9]

This transformation provides the Hessian in the basis of the localized-mode coordinates,

$$\tilde{\boldsymbol{H}}^{sub} = \boldsymbol{U}^T \boldsymbol{H}^{(q),sub}\boldsymbol{U}, \tag{S2}$$

in which the harmonic potential energy surface can be expressed as,

$$V^{\text{harm}}(\tilde{q}_1, \ldots, \tilde{q}_M) = \sum_{i=1}^{M} \frac{1}{2}\tilde{\omega}_i^2\, q_i^2 + \sum_{i<j}^{M} \tilde{H}_{ij}\tilde{q}_i\tilde{q}_j, \tag{S3}$$

where $\tilde{\omega}_i = \sqrt{\tilde{H}_{ii}}$ are the localized-mode frequencies and $\tilde{H}_{ij}$ are the off-diagonal elements of the Hessian with respect to localized modes.

S1.3 L-VSCF/L-VCI

We approximate the anharmonic potential energy surface in localized-mode coordinates as[10,11]

$$V^{\text{1-mode+2h}}(\tilde{q}_1, \cdots, \tilde{q}_M) = \sum_{i=1}^{M} \tilde{V}_i^{(1)}(\tilde{q}_i) + \sum_{i<j}^{M} \tilde{H}_{ij}\tilde{q}_i\tilde{q}_j, \tag{S4}$$

where the anharmonic one-mode potentials $\tilde{V}_i^{(1)}(\tilde{q}_i)$ are calculated on a grid of 16 points per mode[12] and $\tilde{H}_{ij}$ are the off-diagonal elements of the localized-mode Hessian, which describe the harmonic coupling between localized modes.

Now, the vibrational Schrödinger equation can be solved using the L-VSCF/L-VCI ansatz.[12,13] By diagonalizing the L-VCI matrix (including the ground state as well as singly and doubly excited states, i.e., L-VCISD),

$$H_{\boldsymbol{n},\boldsymbol{n}'} = \left\langle \Psi_{\boldsymbol{n}}^{\text{VSCF}} \middle| \hat{H} \middle| \Psi_{\boldsymbol{n}'}^{\text{VSCF}} \right\rangle, \tag{S5}$$

With this, the anharmonic excitation frequencies,

$$\bar{\omega}_n = E_n - E_0, \tag{S6}$$

and transition dipole moments (between ground state and singly excited states as well as between singly and doubly excited states),

$$\boldsymbol{\mu}_{nm} = \left\langle \Psi_n^{\text{L-VCI}} \middle| \hat{\boldsymbol{\mu}} \middle| \Psi_m^{\text{L-VCI}} \right\rangle, \tag{S7}$$

can be calculated.

To illustrate the methodology, the (harmonic) coupling matrices and anharmonic L-VSCF and L-VCI frequencies as well as the corresponding anharmonic shifts for two structures of the dipeptide are listed in Sections S2.1 and S2.2 (Tables S1–S6).

We note that the harmonic coupling can introduce errors in L-VSCF/L-VCISD already at the level of a harmonic potential energy surface [i.e., using Eq. (S3)].[14] While for the dipeptides, these errors are negligible (below 0.05 cm^{-1} in all cases), for polyalanine-10 and polyalanine-15/16 these errors accumulate and become noticeable. Fig. S18 compares the calculated one-dimensional infrared spectra of the polyalanine-10 and polyalanine-15 models. In particular, there is a shift between the harmonic spectra calculated by diagonalizing the

Hessian and those obtained with L-VCIS using the harmonic potential energy surface. This shift is due to the error introduced in L-VCIS by the harmonic coupling as discussed in Ref. 14. This error is also present in the anharmonic spectra. However, it mainly constitutes a constant shift of the vibrational frequencies and should not affect the intensity patterns in the calculated 2D-IR spectra.

S1.4 Calculation of 1D and 2D-IR spectra

One-dimensional (1D) infrared spectra were generated by laying Lorentzian shape functions $f(\bar{\omega}, h = 5)$ over each frequency,

$$f(\bar{\omega}, h) = \frac{h \cdot \text{intensity}_n}{2\pi \cdot \left((\bar{\omega} - \bar{\omega}_n)^2 + (\frac{h}{2})^2\right)}. \tag{S8}$$

The anharmonic frequencies $\bar{\omega}_n$ and transition dipole moments $\boldsymbol{\mu}_{nm}$ are used to generate 2D IR spectra. Generally, 2D IR spectroscopy uses three IR laser pulses to probe third-order nonlinear response. The response functions $R_n(t_1, t_2, t_3)$ can be described by six double-sided Feynman diagrams, representing stimulated emission, ground-state bleaching and excited-state absorption.[11,15–17]

These response functions are then calculated, double Fourier transformed,

$$S_n(\omega_3, t_2, \omega_1) = \int_0^\infty \int_0^\infty \mathrm{i}R_n(t_3, t_2, t_1) e^{\mathrm{i}\omega_1 t_1} e^{\mathrm{i}\omega_3 t_3} \mathrm{d}t_1 \mathrm{d}t_3, \tag{S9}$$

and summed up, in order to get purely absorptive spectra,

$$R(\omega_1, t_2, \omega_3) = \Re\left[\sum_{n=1}^{6} R_n(\omega_1, t_2, \omega_3)\right]. \tag{S10}$$

For further discussion and description of our implementation, we refer to Ref.[11]

S2 Additional data for the dipeptide molecule

S2.1 Additional information for the dipeptide in the α-helix region with the angles $\phi_\alpha = -80$, $\psi_\alpha = -20$

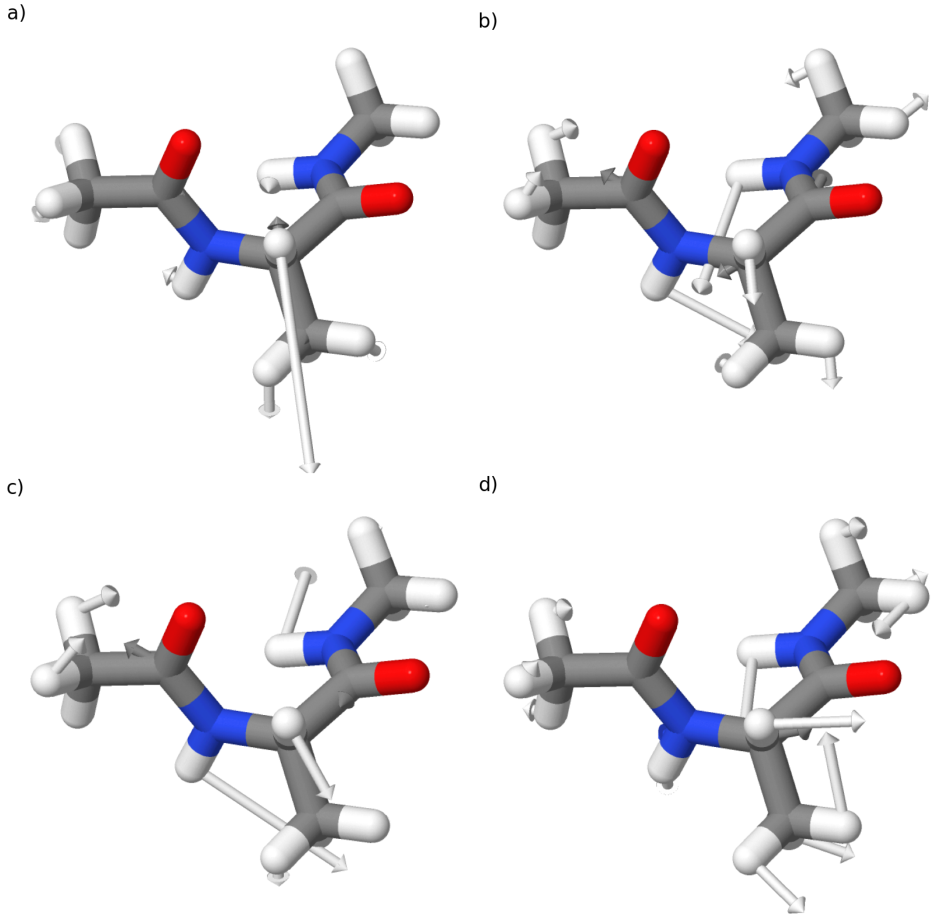

Figure S1: Vibrational normal modes of the dipeptide N-acetyl-L-alanine-N-methylamide in the α-helix region with the angles $\phi_\alpha = -80°$ and $\psi_\alpha = -20°$. (a) shows the bending vibration of C^α-H(1) along the N-C^α bond, (b) shows the bending vibration of C^α-H(1) perpendicular to the N-C^α bond and (c) and (d) show the classical amide III vibrations, that combine N-H bending and C-N stretching vibrations.

Table S1: Coupling matrix of the dipeptide molecule in the α-helix region with the angles $\phi_\alpha = -80$, $\psi_\alpha = -20$ in cm^{-1}.

	Mode 1	Mode 2	Mode 3	Mode 4
Mode 1	1315.74	2.32	7.82	-1.79
Mode 2	2.32	1257.99	17.6	-4.42
Mode 3	7.82	17.6	1215.66	5.6
Mode 4	-1.79	-4.42	5.6	1214.28

Table S2: Local-mode frequencies extracted from the anharmonic L-VSCF calculation for the dipeptide molecule in the α-helix region with the angles $\phi_\alpha = -80$, $\psi_\alpha = -20$ in cm^{-1}.

	Mode 1	Mode 2	Mode 3	Mode 4
$\tilde{\bar{\omega}}_i$	1330.7	1269.7	1219.6	1217.8
$\tilde{\bar{\omega}}_{2i}$	2673.6	2549.2	2443.4	2439.8

The fundamental frequencies for the dipeptide molecule in the α-helix region with the angles $\phi_\alpha = -80$, $\psi_\alpha = -20$ in cm^{-1} from the L-VCI calculations are

$\bar{\omega}_i = \{1208.7, 1222.6, 1274.9, 1331.4\}$

and their corresponding overtones and combination bands are

$\bar{\omega}_{2i,ij} = \{2418.7, 2433.9, 2446.9, 2484.7, 2497.8, 2540.1, 2553.9, 2557.0, 2606.3, 2673.6\}$.

Table S3: Anharmonic shifts Δ_{2i} and Δ_{ij} from the 2D spectrum for each pair of double peaks for the dipeptide molecule in the α-helix region with the angles $\phi_\alpha = -80$, $\psi_\alpha = -20$ in cm^{-1}. The ordering of the table correlates to the location of peaks as seen in the spectrum.

	Mode 1	Mode 2	Mode 3	Mode 4
Mode 4	0.07	0.14	-0.04	-10.76
Mode 3	-1.16	-0.37	-4.2	-0.04
Mode 2	-2.61	-1.66	-0.37	0.14
Mode 1	-1.34	-2.61	-1.16	0.07

S2.2 Additional information for the dipeptide in the β-strand region with the angles $\phi_\beta = -120$, $\psi_\beta = 140$

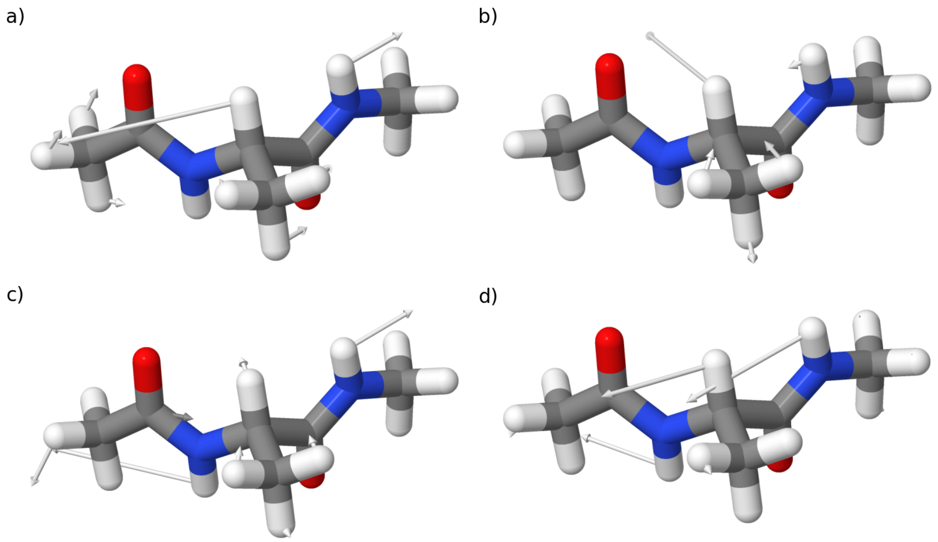

Figure S2: Vibrational normal modes of the dipeptide N-acetyl-L-alanine-N-methylamide in the β-strand region with the angles $\phi_\beta = -120°$ and $\psi_\beta = 140°$. (a) shows the bending vibration of C^α-H(1) along the N-C^α bond, (b) shows the bending vibration of C^α-H(1) perpendicular to the N-C^α bond and (c) and (d) show the classical amide III vibrations, that combine N-H bending and C-N stretching vibrations.

Table S4: Coupling matrix of the dipeptide molecule in the β-strand region with the angles $\phi_\beta = -120$, $\psi_\beta = 140$ in cm^{-1}.

	Mode 1	Mode 2	Mode 3	Mode 4
Mode 1	1299.24	3.76	-7.05	7.97
Mode 2	3.76	1276.52	48.92	23.66
Mode 3	-7.05	48.92	1223.79	0.92
Mode 4	7.97	23.66	0.92	1219.71

Table S5: Local-mode frequencies extracted from the anharmonic L-VSCF calculation for the dipeptide molecule in the β-strand region with the angles $\phi_\beta = -120$, $\psi_\beta = 140$ in cm^{-1}.

	Mode 1	Mode 2	Mode 3	Mode 4
$\tilde{\tilde{\omega}}_i$	1312.2	1289.4	1229.4	1224.5
$\tilde{\tilde{\omega}}_{2i}$	2635.6	2589.3	2463.7	2453.3

The fundamental frequencies for the dipeptide molecule in the β-strand region with the angles $\phi_\beta = -120$, $\psi_\beta = 140$ in cm^{-1} from the L-VCI calculations are

$\bar{\omega}_i = \{1198.6, 1224.2, 1314.2, 1322.5\}$

and their corresponding overtones and combination bands are

$\bar{\omega}_{2i,ij} = \{2398.5, 2423.3, 2449.5, 2511.7, 2524.7, 2537.5, 2546.1, 2633.7, 2639.0, 2649.3\}$.

Table S6: Anharmonic shifts Δ_{2i} and Δ_{ij} from the 2D spectrum for each pair of double peaks for the dipeptide molecule in the β-strand region with the angles $\phi_\beta = -120$, $\psi_\beta = 140$. The ordering of the table correlates to the location of peaks as seen in the spectrum.

	Mode 1	Mode 2	Mode 3	Mode 4
Mode 4	-3.57	0.64	-2.33	-4.23
Mode 3	0.99	0.87	-5.36	-2.33
Mode 2	-0.57	-1.15	0.87	0.64
Mode 1	-1.36	-0.57	0.99	-3.57

S2.3 1D harmonic and anharmonic spectra

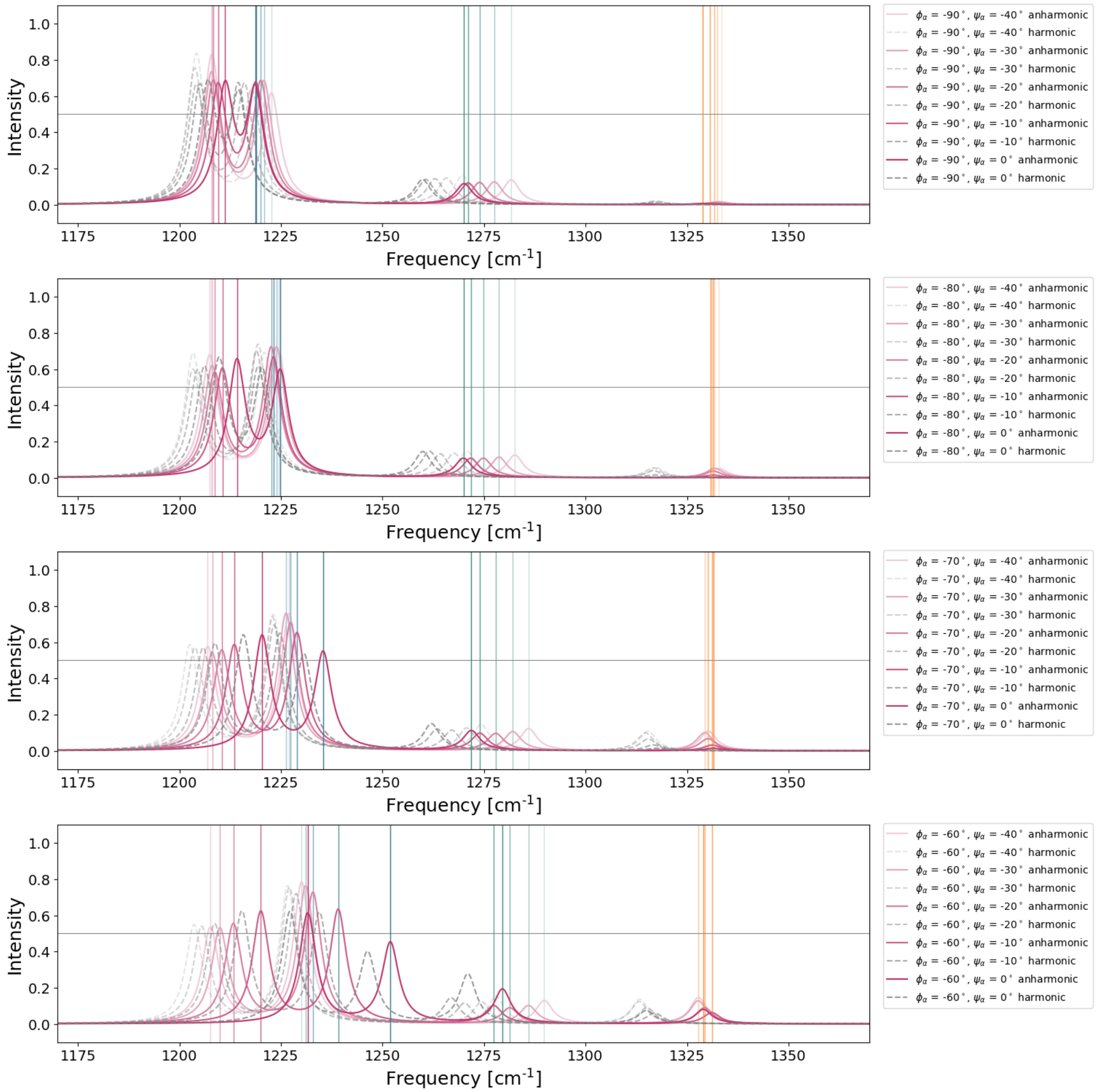

Figure S3: 1D spectra of the N-acetyl-L-alanine-N-methylamide molecule in the α-helix region within the range of 1170 cm^{-1} to 1370 cm^{-1}. Each plot shows the different spectra for varying ψ_α with fixed ϕ_α values. The solid lines are anharmonic spectra, the dashed lines are harmonic spectra. Additionally, vertical lines indicate the frequency of the anharmonic peaks.

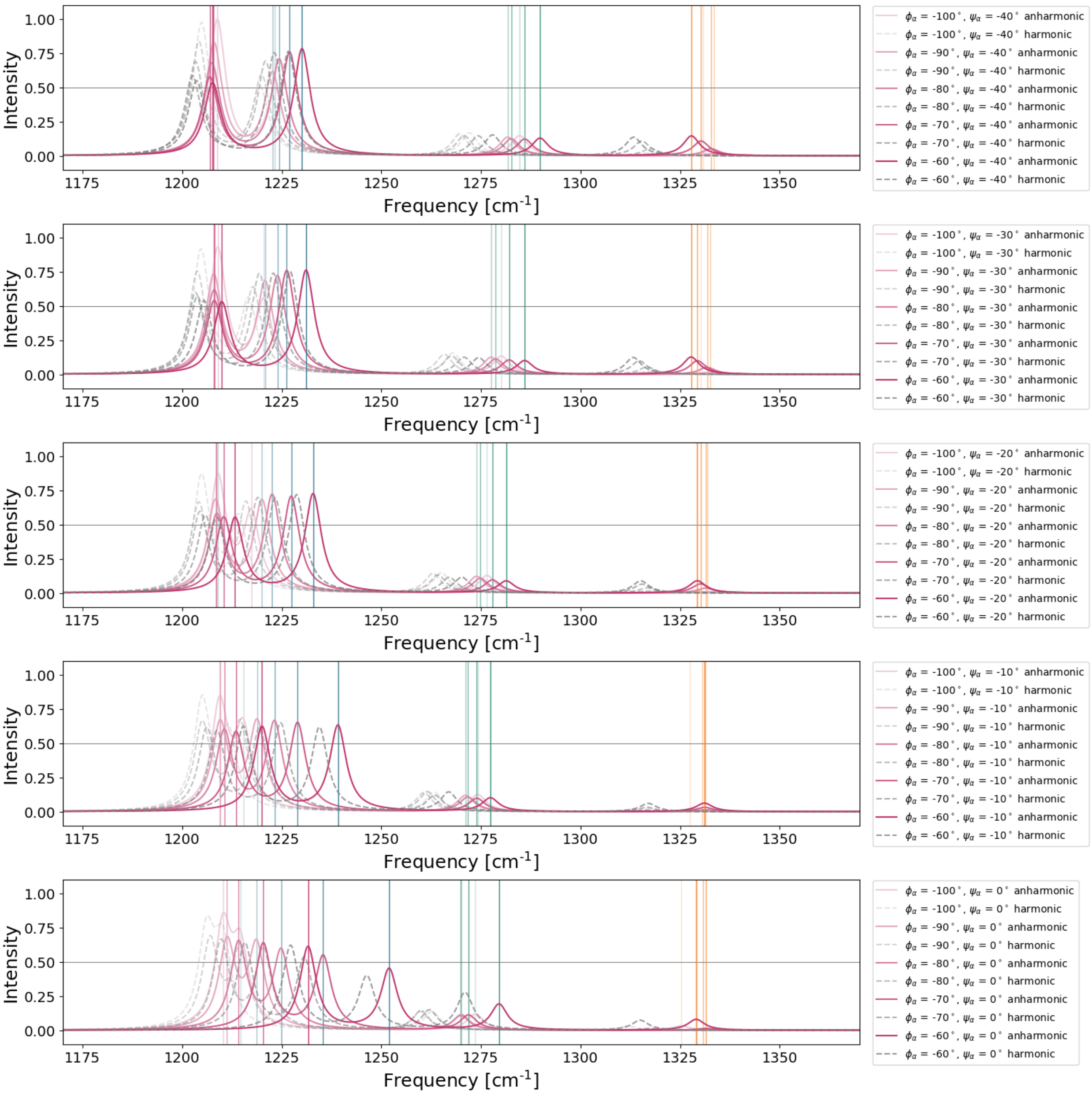

Figure S4: 1D spectra of the N-acetyl-L-alanine-N-methylamide molecule in the α-helix region within the range of 1170 cm^{-1} to 1370 cm^{-1}. Each plot shows the different spectra for varying ϕ_α with fixed ψ_α values. The solid lines are anharmonic spectra, the dashed lines are harmonic spectra. Additionally, vertical lines indicate the frequency of the anharmonic peaks.

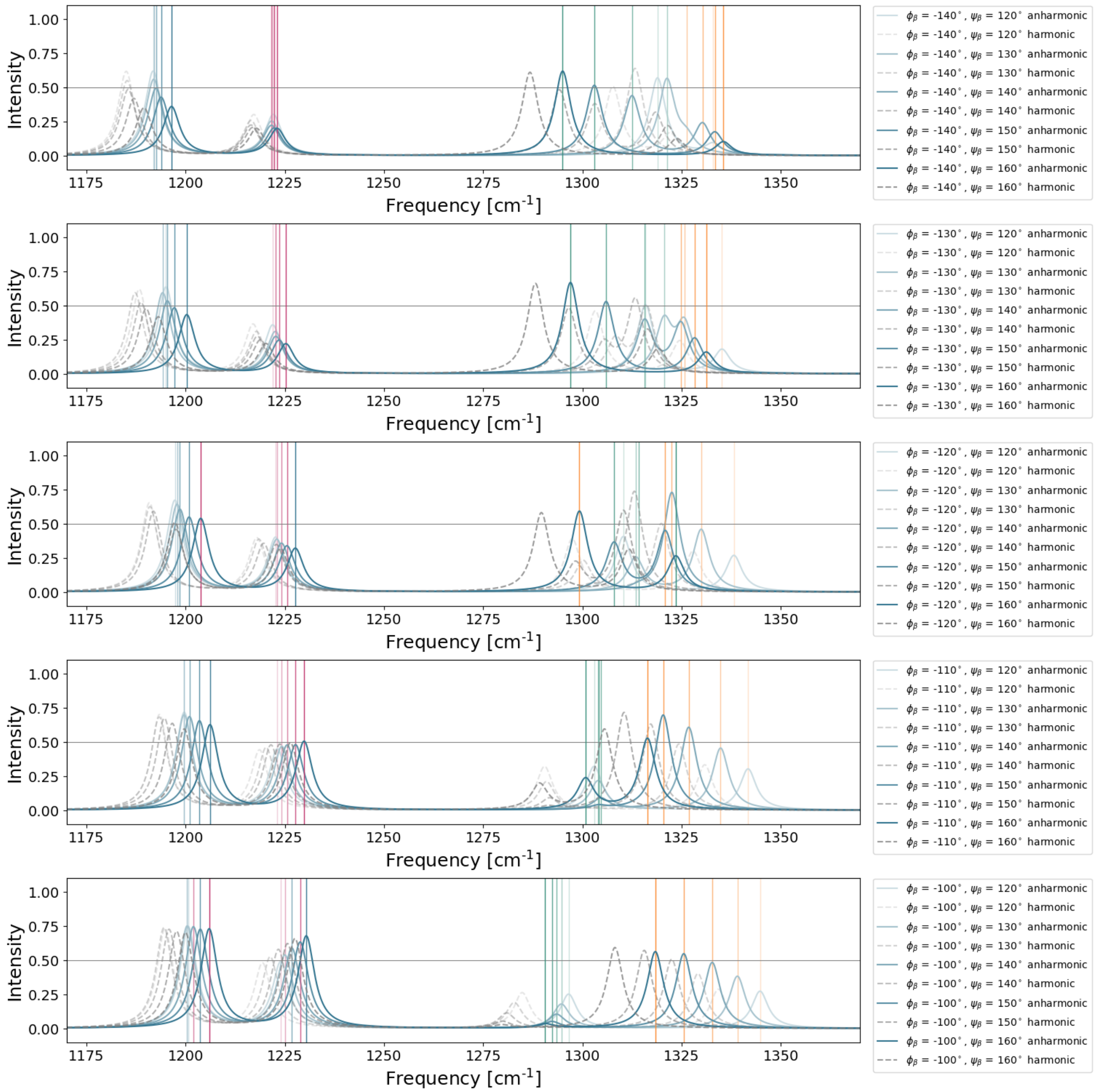

Figure S5: 1D spectra of the N-acetyl-L-alanine-N-methylamide molecule in the β-strand region within the range of 1170 cm^{-1} to 1370 cm^{-1}. Each plot shows the different spectra for varying ψ_β with fixed ϕ_β values. The solid lines are anharmonic spectra, the dashed lines are harmonic spectra. Additionally, vertical lines indicate the frequency of the anharmonic peaks.

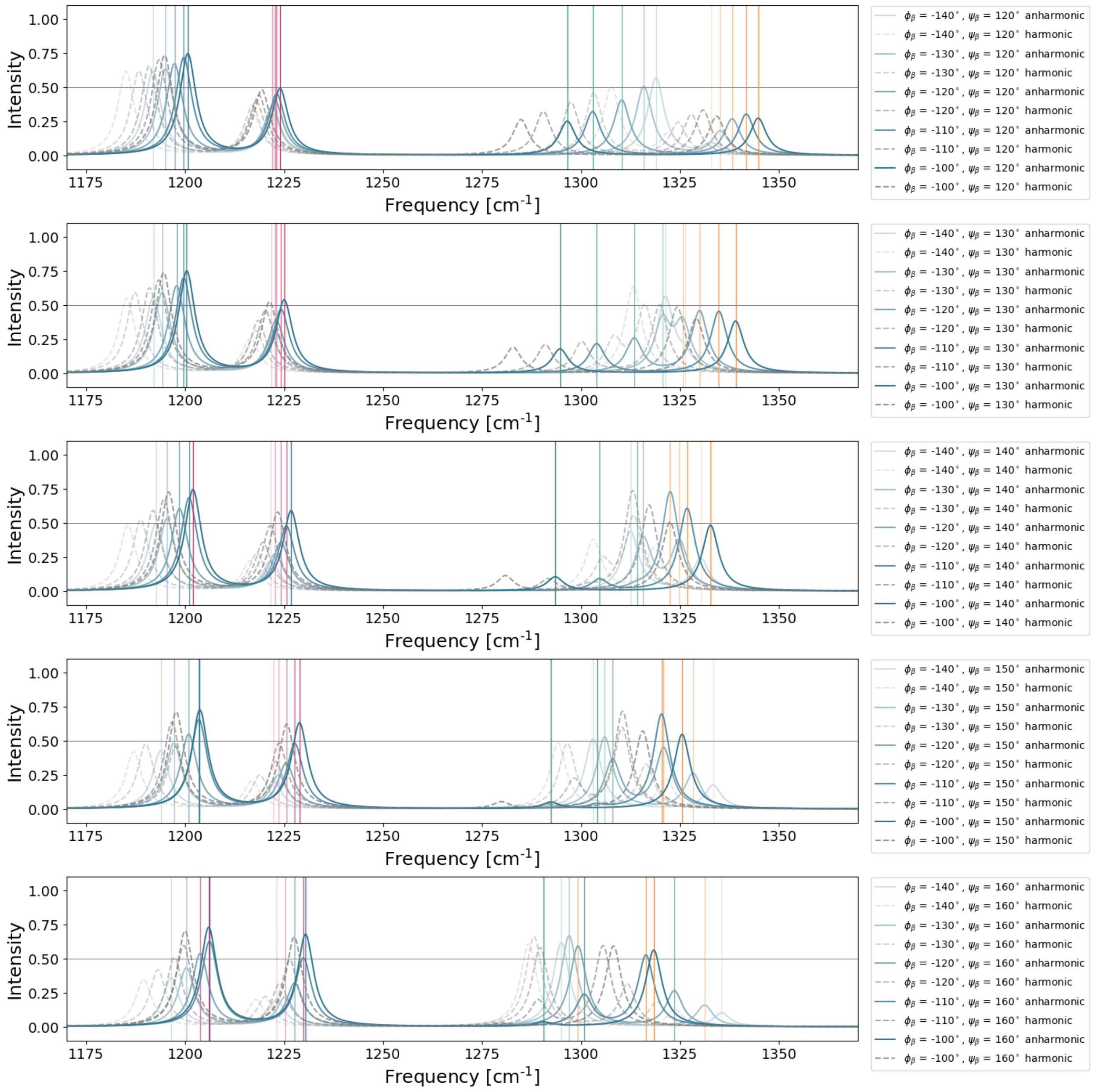

Figure S6: 1D spectra of the N-acetyl-L-alanine-N-methylamide molecule in the β-strand region within the range of 1170 cm^{-1} to 1370 cm^{-1}. Each plot shows the different spectra for varying ϕ_β with fixed ψ_β values. The solid lines are anharmonic spectra, the dashed lines are harmonic spectra. Additionally, vertical lines indicate the frequency of the anharmonic peaks.

S2.4 Mean values of frequencies and coupling matrices for the dipeptide

Table S7: Mean frequency values and standard deviations of the dipeptide molecules.

	Mode 4	Mode 3	Mode 2	Mode 1
α-helix	1212 ± 5.9 cm^{-1}	1225 ± 9.1 cm^{-1}	1278 ± 5.3 cm^{-1}	1330 ± 1.8 cm^{-1}
β-strand	1222 ± 6.9 cm^{-1}	1202 ± 10.5 cm^{-1}	1306 ± 9.7 cm^{-1}	1329 ± 9.3 cm^{-1}

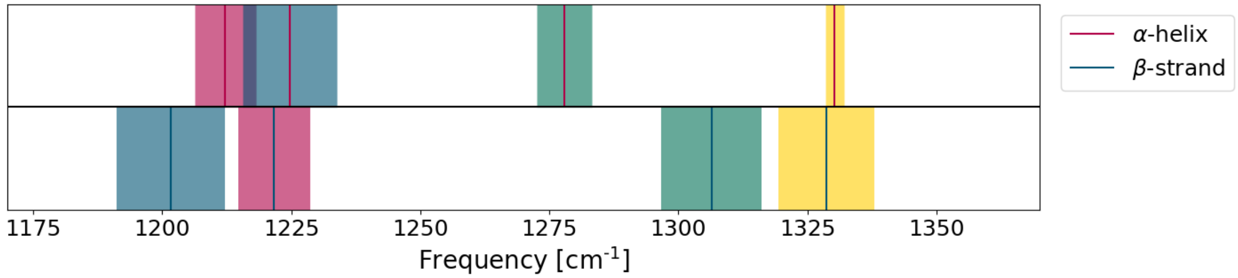

Figure S7: Mean frequency values and standard deviations in cm^{-1} of the dipeptide molecules as shown in Table S7.

Table S8: Mean coupling matrix of the dipeptide in the α-helix region with corresponding standard deviations in cm^{-1}.

	Mode 1	Mode 2	Mode 3	Mode 4
Mode 1	1310.19 ± 13.72	2.7 ± 1.37	5.16 ± 6.78	0.15 ± 6
Mode 2	2.7 ± 1.37	1265.04 ± 15.15	14.14 ± 6.14	-3.56 ± 6.27
Mode 3	5.16 ± 6.78	14.14 ± 6.14	1218.39 ± 6.73	6.02 ± 2.78
Mode 4	0.15 ± 6	-3.56 ± 6.27	6.02 ± 2.78	1216.79 ± 7.09

Table S9: Mean coupling matrix of the dipeptide in the β-strand region with corresponding standard deviations in cm^{-1}.

	Mode 1	Mode 2	Mode 3	Mode 4
Mode 1	1276.81 ± 12.72	6.49 ± 3.49	35.19 ± 20.44	9.84 ± 24.91
Mode 2	6.49 ± 3.49	1297.17 ± 13.82	6.05 ± 17.37	5.86 ± 16.08
Mode 3	35.19 ± 20.44	6.05 ± 17.37	1224.97 ± 2.24	1.96 ± 1.4
Mode 4	9.84 ± 24.91	5.86 ± 16.08	1.96 ± 1.4	1219.65 ± 3.27

S2.5 Overview of all 2D spectra and their anharmonicities of fundamental modes

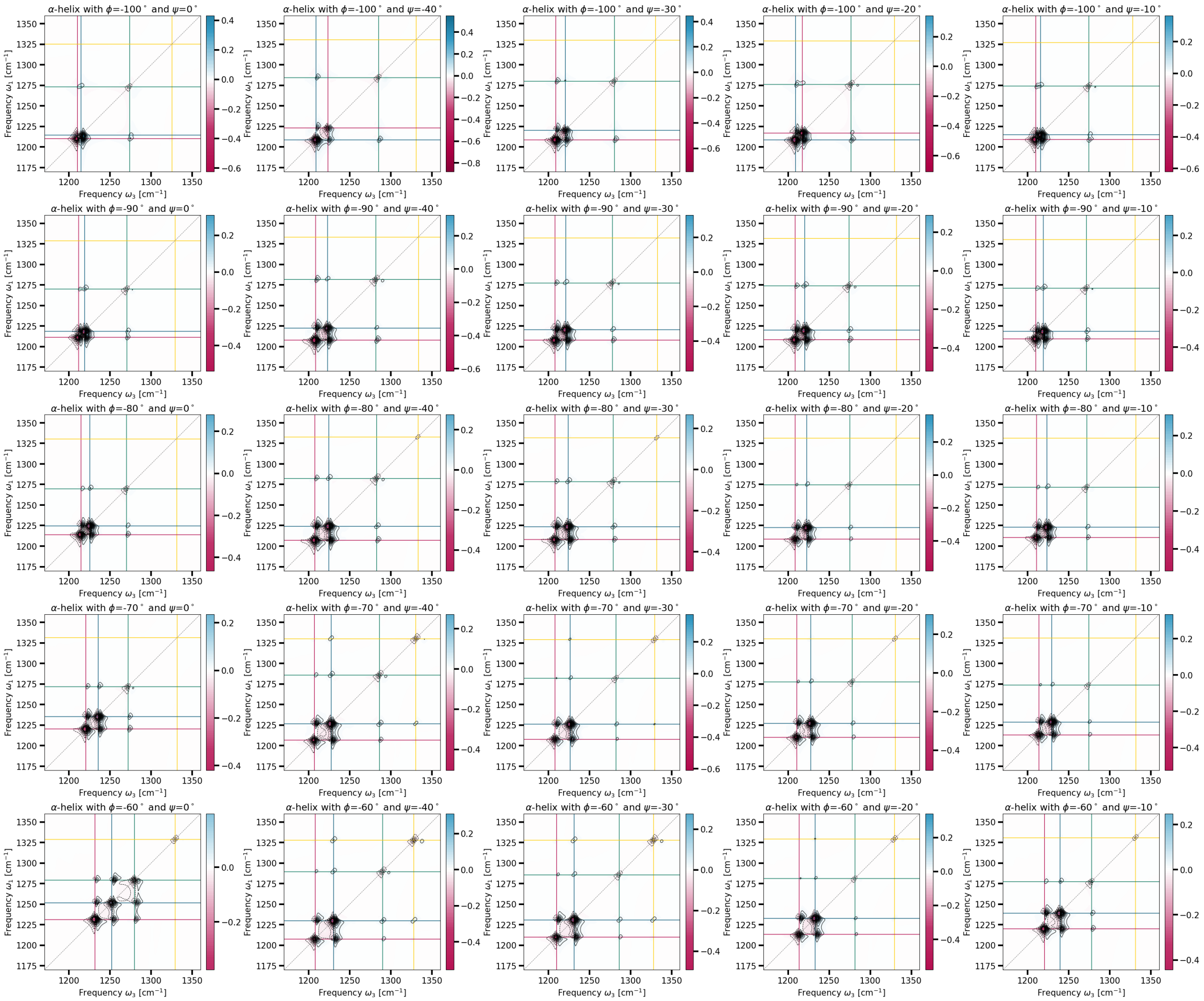

Figure S8: All calculated 2D spectra of the N-acetyl-L-alanine-N-methylamide molecule in the α-helix region.

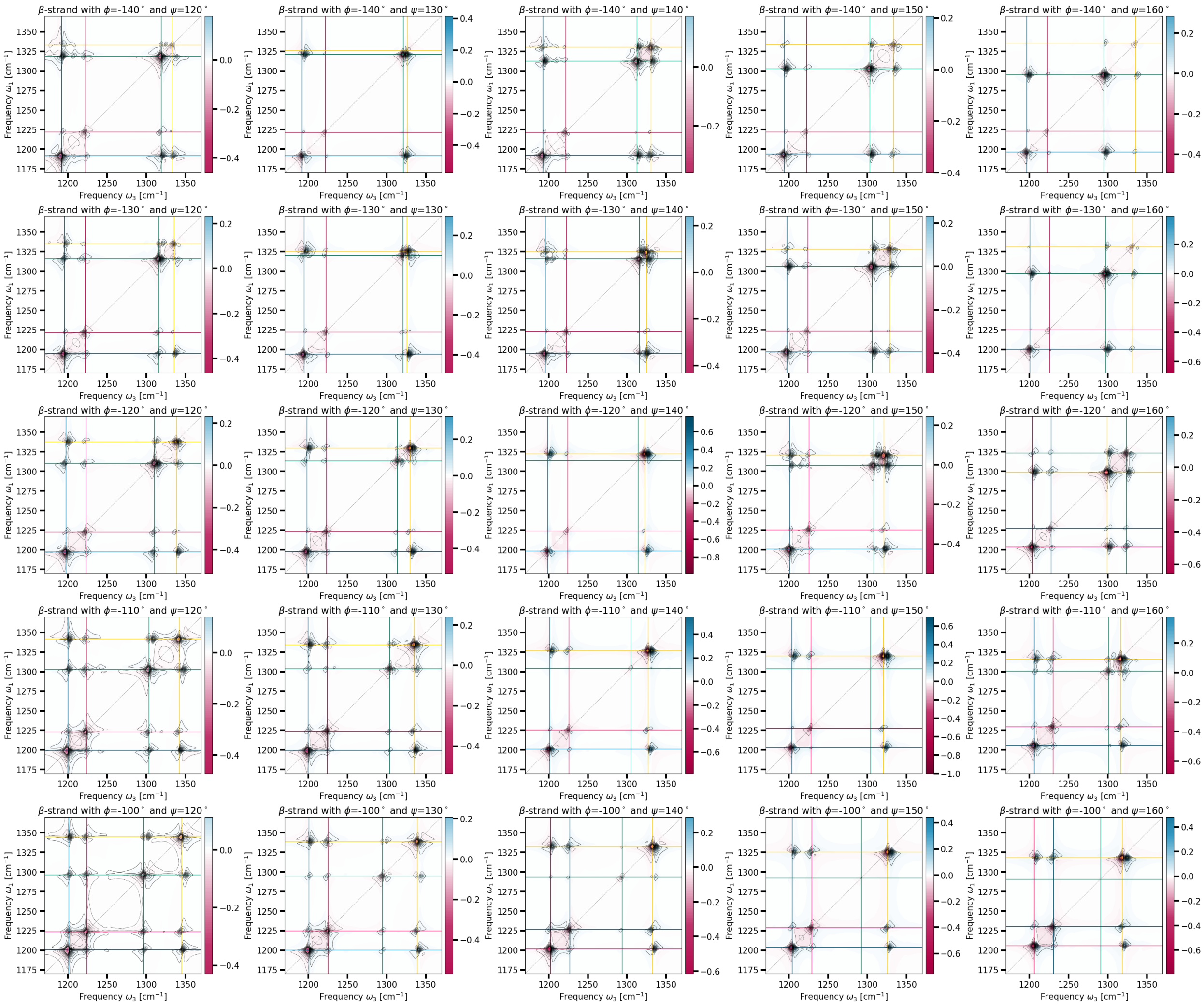

Figure S9: All calculated 2D spectra of the N-acetyl-L-alanine-N-methylamide molecule in the β-strand region.

S3 Additional data for the polyalanine molecules

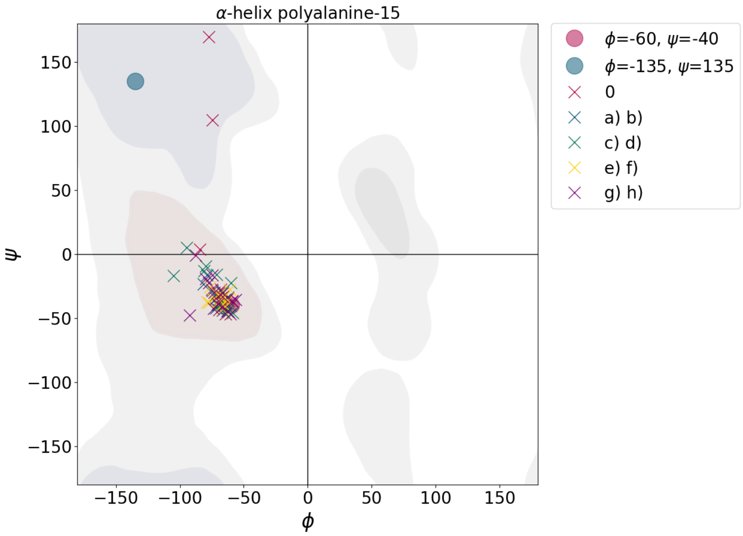

Figure S10: Ramachandran plot with all found backbone angles of the polyalanine molecules in the α-helix region. The round dots show the angles of the polyalanine-10 molecules in the α-helix region (red) and the β-strand region (blue). The red crosses (0) show the backbone angles of the polyalanine-15 molecule shown in figure 3(b). The other crosses show the backbone angles of the polyalaine-15 molecules shown in figure S15.

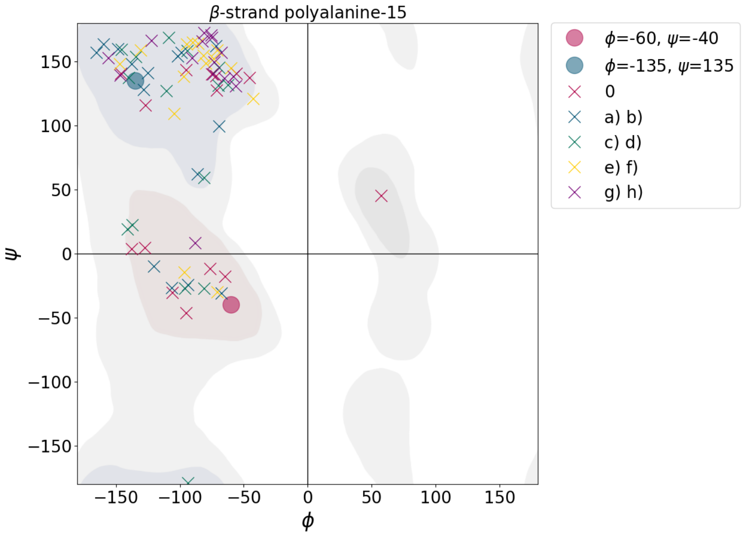

Figure S11: Ramachandran plot with all found backbone angles of the polyalanine molecules in the β-strand region. The round dots show the angles of the polyalanine-10 molecules in the α-helix region (red) and the β-strand region (blue). The red crosses (0) show the backbone angles of the polyalanine-15 molecule shown in Fig. 3b. The other crosses show the backbone angles of the polyalaine-15 molecules shown in figure S16.

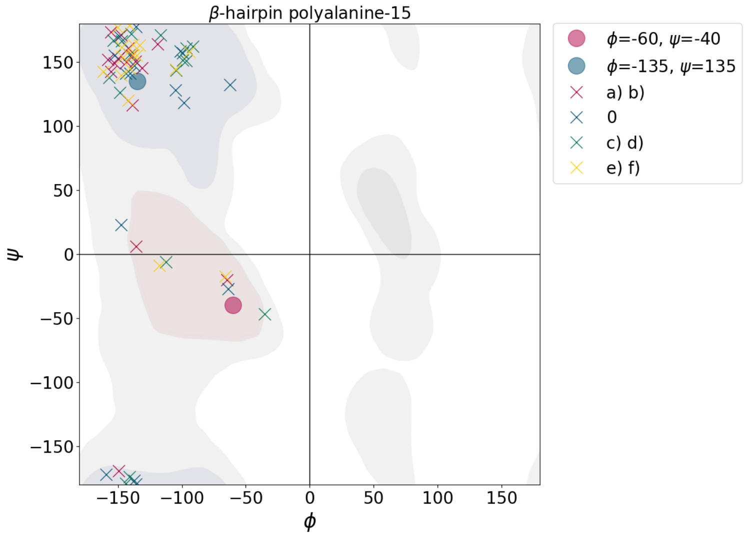

Figure S12: Ramachandran plot with all found backbone angles of the polyalanine molecules in the β-hairpin region. The round dots show the angles of the polyalanine-10 molecules in the α-helix region (red) and the β-strand region (blue). The blue crosses (0) show the backbone angles of the polyalanine-16 molecule shown in Fig. 3b. The other crosses show the backbone angles of the polyalaine-16 molecules shown in figure S17.

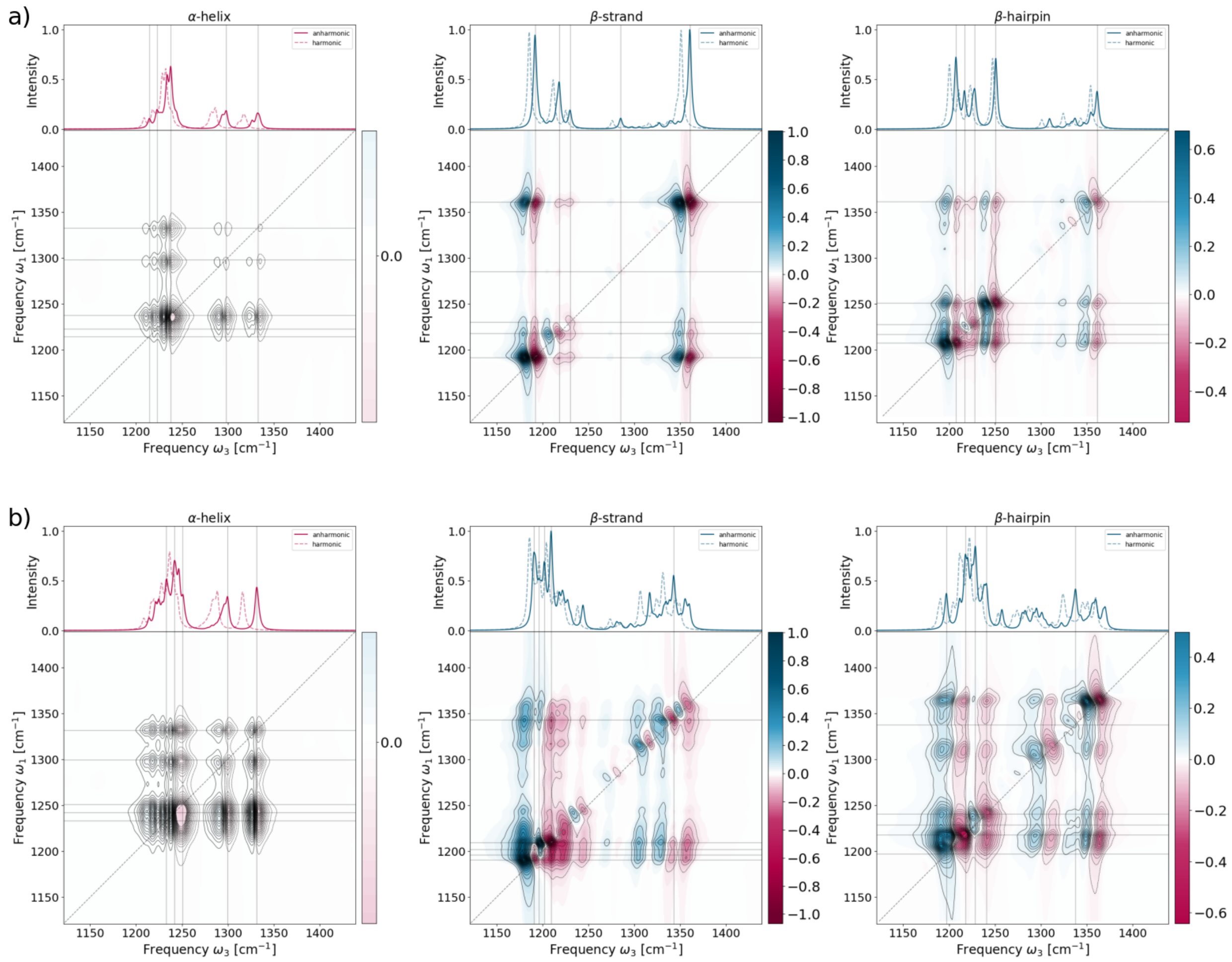

Figure S13: Calculated 2D-IR spectra (L-VSCF/L-VCI) of (a) polyalanine-10 in an idealized α-helical and an idealized β-strand conformation and of (b) a snapshot of polyalanine-15 extracted from a molecular dynamics simulation of an α-helix and a β-strand for the $\langle ZZXX \rangle$ polarization condition. In the insets above the 2D-IR spectra, the calculated harmonic and anharmonic infrared spectra are shown for comparison. Horizontal and vertical lines indicate the five largest peaks of the 1D spectra.

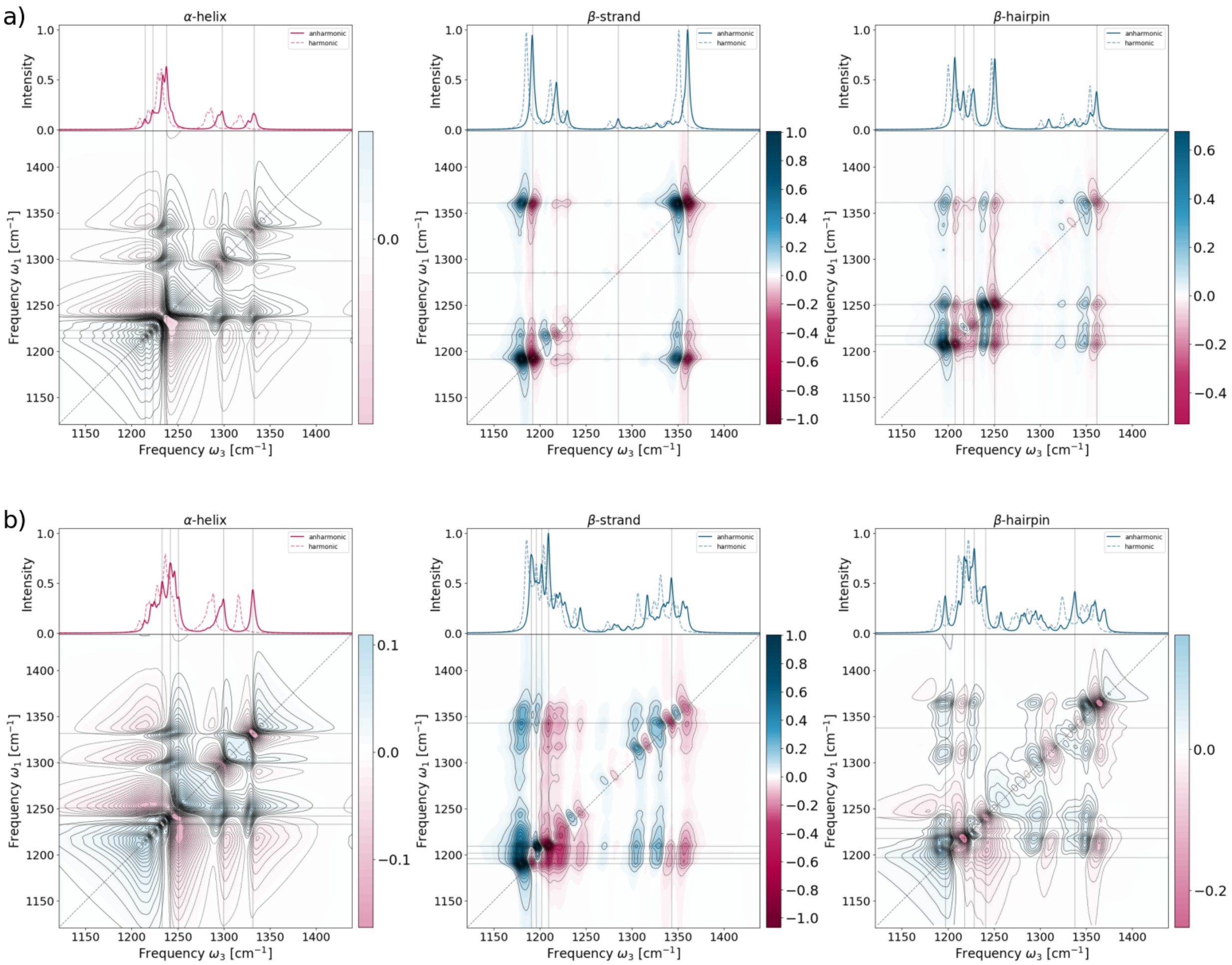

Figure S14: Calculated 2D-IR spectra (L-VSCF/L-VCI) of (a) polyalanine-10 in an idealized α-helical and an idealized β-strand conformation and of (b) a snapshot of polyalanine-15 extracted from a molecular dynamics simulation of an α-helix and a β-strand for the $\langle ZXXZ \rangle$ polarization condition. In the insets above the 2D-IR spectra, the calculated harmonic and anharmonic infrared spectra are shown for comparison. Horizontal and vertical lines indicate the five largest peaks of the 1D spectra.

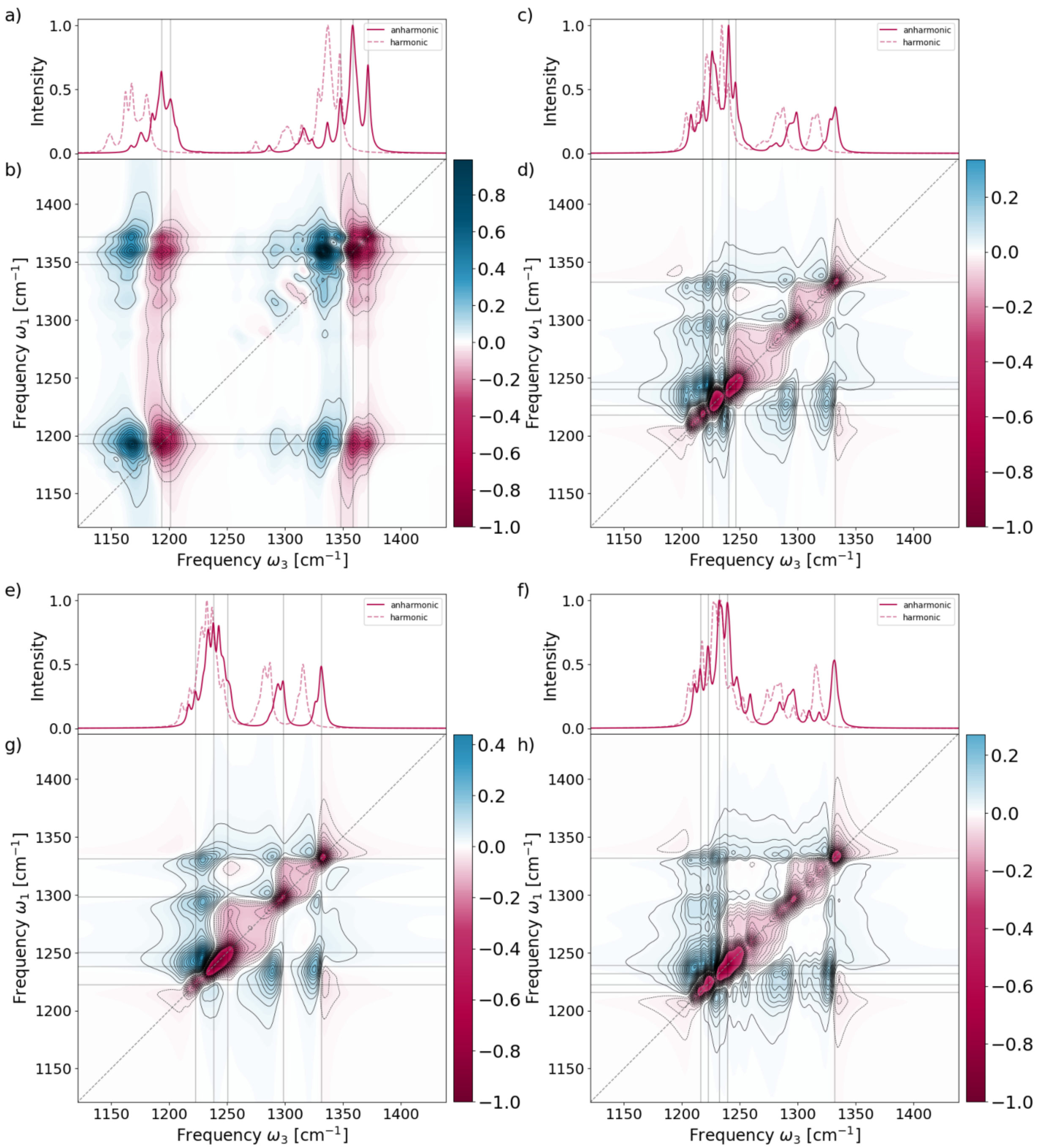

Figure S15: Calculated 2D-IR spectra (L-VSCF/L-VCI) of four additional snapshots of polyalanine-15 extracted from a molecular dynamics simulation of an α-helix. In the insets above the 2D-IR spectra, the calculated harmonic and anharmonic infrared spectra are shown for comparison. In each plot the maximum is set to one.

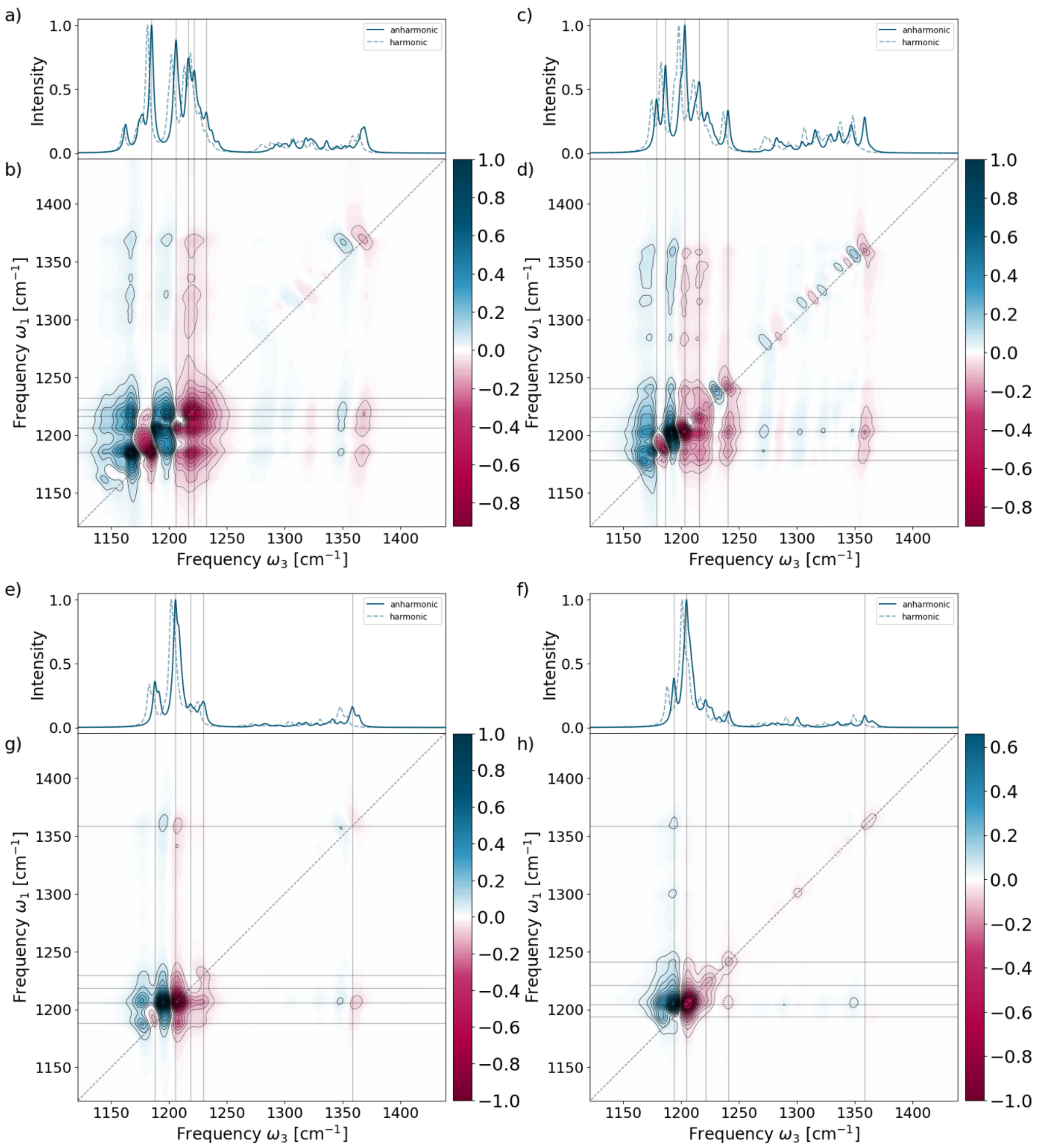

Figure S16: Calculated 2D-IR spectra (L-VSCF/L-VCI) of four additional snapshots of polyalanine-15 extracted from a molecular dynamics simulation of an β-strand. In the insets above the 2D-IR spectra, the calculated harmonic and anharmonic infrared spectra are shown for comparison. In each plot the maximum is set to one.

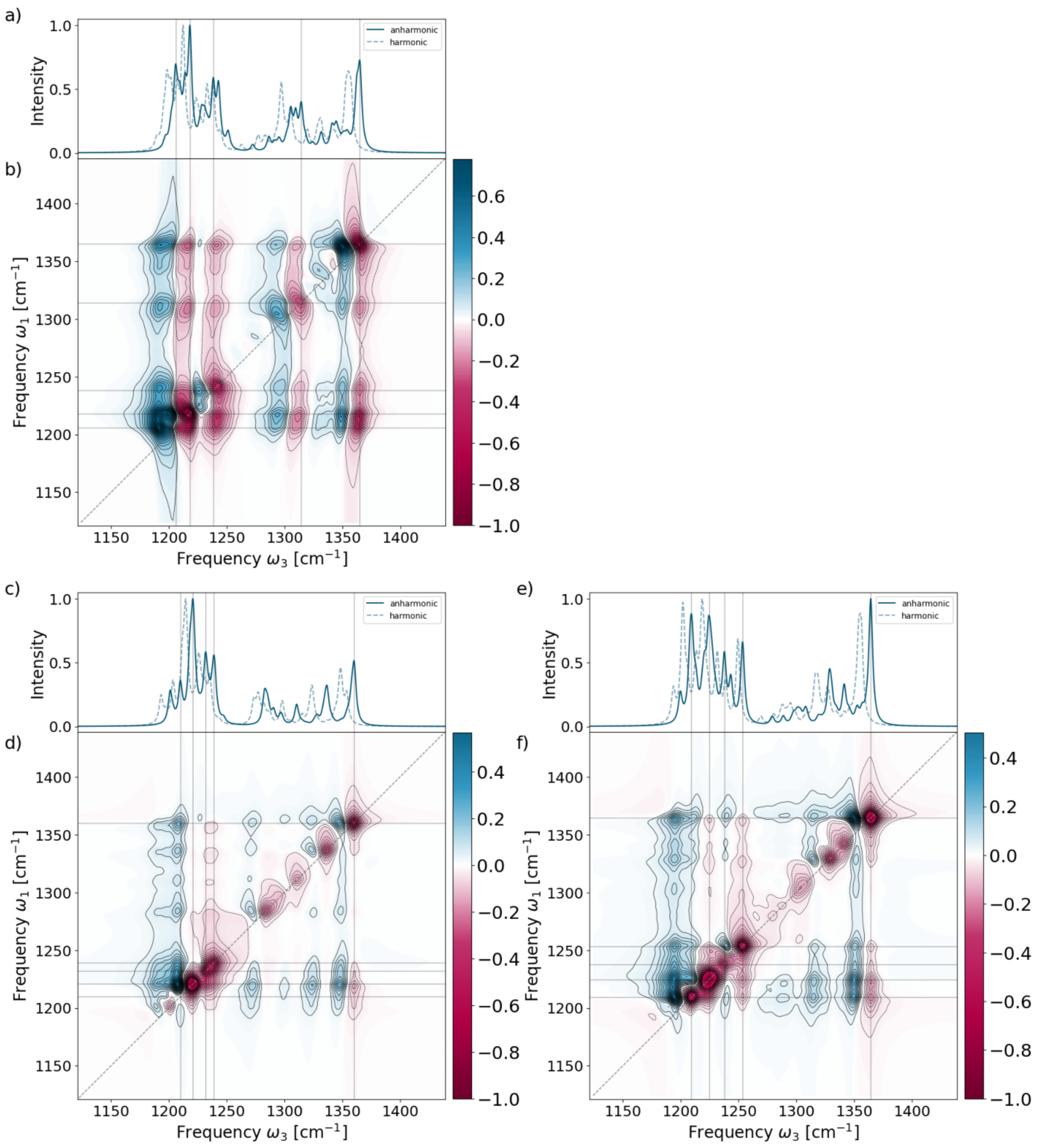

Figure S17: Calculated 2D-IR spectra (L-VSCF/L-VCI) of three additional snapshots of polyalanine-15 extracted from a molecular dynamics simulation of an β-turn. In the insets above the 2D-IR spectra, the calculated harmonic and anharmonic infrared spectra are shown for comparison. In each plot the maximum is set to one.

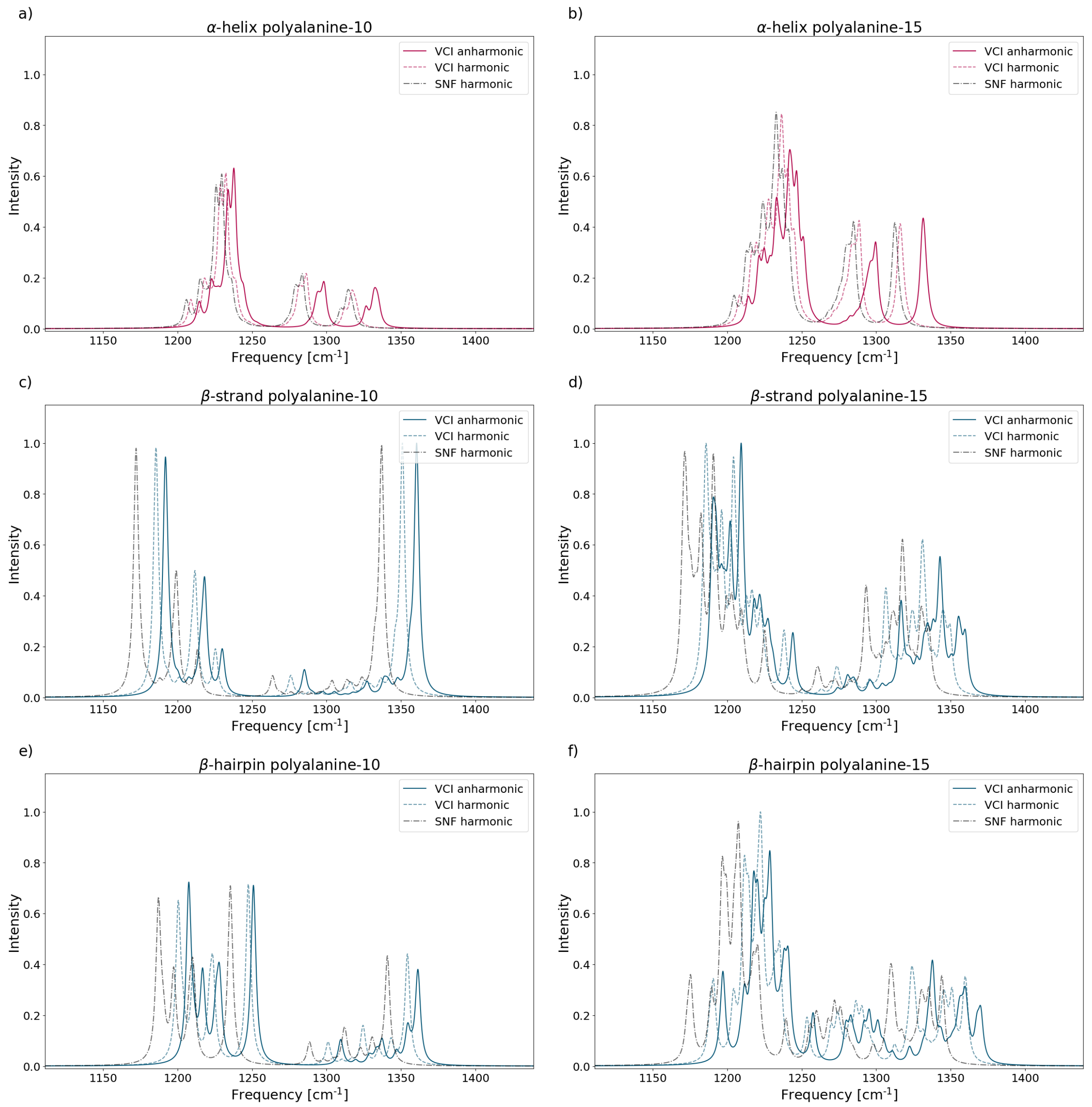

Figure S18: Comparison of 1D spectra of the polyalanine molecules in the range of 1110 cm^{-1} to 1440 cm^{-1}. In the left column the spectra for polyalanine-10 in the α-helix region (a), β-strand region (c) and the β-hairpin (e) are shown, in the right column the spectra for polyalanine-15 in the α-helix region (b), β-strand region (d) and the β-hairpin (f) are shown. The solid colored lines show the results from the L-VSCF/L-VCI calculations, the dashed colored lines show the results from the L-VSCF/L-VCI calculations with harmonic potentials and the dashed/dotted gray lines show the results from the harmonic calculations using Turbomole and SNF.

Table S10: Mode contributions for polyalanine-10 in the α-helix configuration in percent for each vibrational mode n_{mode} and their corresponding frequencies in cm^{-1} and intensities in km/mol. As a subset we chose the modes 184–214. The modes 184–194 include the classical amide III vibrations, the modes 195–204 are the C^{α}-H(2) vibrations and the modes 205–214 are the C^{α}-H(1) vibrations.

n_{mode}	Frequency	Intensity	N	C	O	H
180	1146.4	15.8031	20.8	45.0	3.3	30.9
181	1147.6	11.0207	20.5	45.9	2.6	31.0
182	1147.7	16.1920	20.7	44.3	3.7	31.3
183	1150.0	20.8034	19.3	41.8	3.5	35.4
184	1205.9	26.7601	4.8	41.6	3.8	49.8
185	1215.0	38.0876	6.5	44.6	3.8	45.1
186	1217.1	10.6581	5.2	44.2	3.7	46.8
187	1219.3	11.4426	5.2	45.6	3.1	46.2
188	1222.2	9.9292	4.9	45.9	2.5	46.7
189	1225.7	121.4587	5.0	46.7	2.1	46.3
190	1229.8	135.8764	4.9	47.8	1.7	45.6
191	1233.5	14.0254	5.1	49.4	1.6	43.9
192	1236.3	31.3765	4.9	49.8	1.5	43.8
193	1240.0	0.9302	5.7	52.9	1.3	40.2
194	1245.4	1.4810	5.5	52.6	1.3	40.6
195	1273.0	1.8322	1.1	29.1	1.2	68.7
196	1274.5	1.8480	1.1	29.5	1.2	68.3
197	1276.6	1.4853	1.1	30.4	1.4	67.1
198	1278.0	7.0084	1.0	30.2	1.7	67.2
199	1278.9	22.6116	0.8	29.7	1.9	67.6
200	1280.0	6.8252	0.7	29.5	2.1	67.6
201	1280.9	6.8391	0.7	29.2	2.4	67.7
202	1282.1	3.2178	0.8	28.7	2.8	67.8
203	1283.1	7.3334	1.0	28.7	2.5	67.7
204	1283.9	42.6066	0.8	28.4	3.0	67.7
205	1309.3	15.5115	1.7	16.5	1.3	80.5
206	1313.8	16.9449	2.6	16.2	0.8	80.4
207	1314.6	10.6041	2.5	16.8	1.2	79.5
208	1315.0	4.6557	2.5	16.7	1.4	79.4
209	1315.9	7.4603	2.4	17.0	1.6	79.0
210	1316.8	5.6447	2.4	17.3	1.9	78.4
211	1317.5	8.2840	2.4	17.9	2.2	77.5
212	1318.2	0.2761	2.0	18.7	2.6	76.7
213	1319.1	0.5128	1.8	19.2	3.0	76.0
214	1320.9	0.2199	2.0	19.5	3.0	75.5
215	1350.4	25.7384	0.1	20.3	0.1	79.5
216	1358.0	16.7683	0.1	19.6	0.1	80.2
217	1359.9	15.3638	0.2	19.6	0.0	80.2
218	1360.4	14.4525	0.2	19.5	0.0	80.3

Table S11: Mode contribution for polyalanine-10 in the β-strand configuration in percent for each vibrational mode n_{mode} and their corresponding frequencies in cm^{-1} and intensities in km/mol. As a subset we chose the modes 184–214. The modes 184–194 include the classical amide III vibrations, the modes 195–204 are the C^{α}-H(2) vibrations and the modes 205–214 are the C^{α}-H(1) vibrations.

n_{mode}	Frequency	Intensity	N	C	O	H
180	1134.2	4.1043	20.3	40.0	3.6	36.1
181	1138.6	0.8374	21.0	38.4	3.9	36.7
182	1141.2	104.6796	21.6	36.8	4.2	37.4
183	1144.5	75.9424	23.2	40.4	3.1	33.3
184	1172.1	262.4955	12.7	31.0	2.4	53.9
185	1173.2	3.3535	12.3	30.6	2.5	54.6
186	1175.0	12.7379	11.7	29.9	2.7	55.7
187	1177.4	0.2518	11.0	29.6	2.9	56.4
188	1180.4	9.6749	10.4	30.1	3.0	56.5
189	1183.9	0.6181	9.8	31.4	3.1	55.8
190	1188.0	9.6939	9.2	33.5	3.0	54.4
191	1192.4	4.1419	8.7	36.2	2.9	52.2
192	1196.5	25.1251	8.6	39.0	2.8	49.6
193	1199.1	125.9123	8.9	41.1	2.6	47.4
194	1213.4	48.7128	2.3	40.6	3.9	53.2
195	1263.8	22.5338	4.2	22.2	1.9	71.7
196	1269.1	2.6309	3.9	25.2	1.8	69.0
197	1276.1	4.1926	3.6	28.1	1.7	66.6
198	1283.5	4.5272	3.0	29.7	1.6	65.7
199	1290.0	1.9371	2.3	29.3	1.3	67.1
200	1294.7	2.3464	1.6	27.3	1.0	70.1
201	1297.4	1.2749	1.1	25.0	0.7	73.1
202	1298.8	0.9696	0.9	23.4	0.6	75.1
203	1299.6	2.6558	0.7	22.5	0.5	76.3
204	1303.7	15.3893	0.2	29.4	1.1	69.3
205	1313.4	12.8977	2.2	20.2	0.2	77.4
206	1315.8	3.6609	1.4	22.2	0.3	76.1
207	1316.4	0.4728	1.6	22.8	0.4	75.2
208	1317.6	5.5063	1.9	24.4	0.6	73.1
209	1320.0	0.3758	2.3	27.1	0.9	69.7
210	1323.6	12.7009	2.7	30.6	1.2	65.4
211	1328.2	0.2917	2.9	33.5	1.5	62.2
212	1332.3	35.6634	2.8	34.8	1.6	60.8
213	1335.2	0.0930	2.7	35.1	1.6	60.6
214	1337.0	268.5280	2.7	35.1	1.6	60.7
215	1344.9	40.2886	0.3	22.0	0.1	77.6
216	1353.0	8.5791	0.1	19.9	0.1	80.0
217	1354.4	10.7214	0.1	18.9	0.0	81.0
218	1354.6	5.9538	0.0	18.6	0.0	81.3

Table S12: Mode contributions for polyalanine-10 in the β-hairpin configuration in percent for each vibrational mode $n_{\mathbf{mode}}$ and their corresponding frequencies in cm^{-1} and intensities in km/mol. As a subset we chose the modes 204–235. The modes 204–219 include the classical amide III vibrations, the modes 220–227 are the C$^{\alpha}$-H(2) vibrations and the modes 228–235 are the C$^{\alpha}$-H(1) vibrations.

n_{mode}	Frequency	Intensity	H	C	O	N
201	1142.7	3.3772	32.4	43.4	2.5	21.7
202	1146.9	1.0500	29.2	44.8	1.6	24.5
203	1147.2	193.4727	29.9	44.2	1.7	24.2
204	1187.1	168.2582	59.1	27.4	3.4	10.0
205	1188.4	3.3949	59.8	27.0	3.7	9.5
206	1190.1	3.4625	59.1	27.6	3.3	10.0
207	1190.4	26.3127	59.5	27.8	3.3	9.4
208	1197.3	53.5777	53.3	30.4	3.1	13.2
209	1197.4	41.5543	53.3	30.4	3.1	13.2
210	1206.5	0.3495	51.4	32.4	3.0	13.2
211	1208.1	49.2024	49.5	35.6	2.7	12.2
212	1210.3	56.7180	48.4	36.8	2.7	12.0
213	1210.5	33.8924	50.0	33.3	2.9	13.8
214	1235.4	147.4727	48.2	41.9	4.2	5.7
215	1235.6	47.6745	48.0	42.1	4.2	5.7
216	1288.6	24.9413	68.7	26.2	2.4	2.7
217	1290.6	0.0690	69.5	25.5	2.3	2.7
218	1297.8	4.3105	70.1	26.3	1.6	2.1
219	1297.9	0.3862	69.4	26.9	1.6	2.1
220	1303.8	1.8664	76.9	21.9	0.6	0.6
221	1304.1	1.4867	77.0	21.8	0.6	0.6
222	1305.5	1.8993	78.1	20.2	0.7	1.0
223	1307.3	0.1038	79.3	18.9	0.6	1.1
224	1310.5	0.0467	66.9	30.0	2.1	1.0
225	1311.3	14.7402	67.1	29.8	2.1	1.0
226	1312.4	24.3665	76.6	20.8	0.2	2.4
227	1312.5	4.2168	76.7	20.8	0.3	2.2
228	1322.7	15.5362	70.1	27.2	1.2	1.5
229	1325.0	0.1354	69.4	27.8	1.4	1.5
230	1329.8	0.1921	68.1	29.2	1.2	1.4
231	1330.6	25.3223	67.9	29.3	1.4	1.4
232	1336.8	2.5753	67.1	29.5	1.4	2.0
233	1338.2	0.0917	70.0	26.9	1.3	1.8
234	1340.5	1.3318	67.6	29.2	1.3	1.8
235	1340.9	116.6341	71.3	26.0	1.2	1.5
236	1346.2	35.6345	77.5	22.1	0.1	0.3
237	1346.3	38.0095	77.5	22.1	0.1	0.3
238	1350.8	5.6498	79.1	20.6	0.1	0.2

Table S13: Mode contributions for polyalanine-15 in the α-helix configuration in percent for each vibrational mode $n_{\mathbf{mode}}$ and their corresponding frequencies in cm^{-1} and intensities in km/mol. As a subset we chose the modes 263–314. The modes 263–284 include the classical amide III vibrations, the modes 285–299 are the C^{α}-H(2) vibrations and the modes 300–314 are the C^{α}-H(1) vibrations.

n_{mode}	Frequency	Intensity	H	C	O	N
260	1114.4	2.3741	33.7	59.3	1.0	6.0
261	1115.5	3.7162	32.8	58.3	1.0	7.9
262	1116.0	6.3240	32.1	58.8	1.2	7.9
263	1116.8	1.9569	29.8	57.1	1.3	11.8
264	1118.3	4.8328	29.1	57.2	1.4	12.4
265	1118.7	5.2395	29.7	57.2	1.4	11.8
266	1120.2	11.6298	29.9	57.3	1.3	11.6
267	1120.5	2.5371	29.4	57.4	1.3	12.0
268	1122.2	4.5876	29.2	57.1	1.3	12.4
269	1134.9	62.2036	48.7	33.4	1.9	16.0
270	1138.9	72.1236	35.3	47.4	0.8	16.5
271	1140.0	16.2370	31.8	50.3	0.4	17.6
272	1143.6	12.1664	31.9	49.3	0.3	18.5
273	1145.3	2.7953	32.9	49.2	0.5	17.4
274	1146.5	12.0373	33.8	49.8	0.8	15.6
275	1148.1	12.6703	37.3	47.5	1.3	13.8
276	1148.4	0.0879	35.4	48.7	0.9	15.0
277	1150.7	6.4288	35.9	49.0	1.2	13.9
278	1151.7	35.9056	36.6	48.5	1.3	13.5
279	1153.1	24.5265	37.6	48.2	1.5	12.6
280	1153.8	4.4413	39.4	48.1	2.2	10.3
281	1154.2	10.7606	38.7	47.6	1.8	12.0
282	1155.1	8.8472	39.6	46.8	1.9	11.7
283	1155.7	0.6899	39.7	47.6	2.1	10.5
284	1174.0	0.6655	35.8	48.9	1.1	14.2
285	1247.7	14.6954	66.2	32.0	0.1	1.8
286	1260.2	0.6838	65.3	34.3	0.1	0.3
287	1265.3	1.0950	66.0	32.9	0.4	0.7
288	1267.4	1.6253	66.0	32.8	0.5	0.8
289	1267.7	0.8339	66.3	32.5	0.5	0.7
290	1269.5	3.6859	67.4	31.0	0.6	1.0

Table S14: Mode contributions for polyalanine-15 in the α-helix configuration in percent for each vibrational mode $n_{\mathbf{mode}}$ and their corresponding frequencies in cm^{-1} and intensities in km/mol (continued).

n_{mode}	Frequency	Intensity	H	C	O	N
291	1269.7	2.2993	68.5	30.6	0.6	0.4
292	1270.3	5.7602	67.3	31.3	0.7	0.8
293	1270.5	7.1534	66.7	31.8	0.6	0.8
294	1273.1	6.8729	67.9	30.5	0.9	0.7
295	1274.0	4.6626	68.4	29.8	1.0	0.9
296	1274.3	8.7481	65.6	31.7	0.9	1.7
297	1275.8	9.5076	67.4	30.4	1.1	1.0
298	1277.1	15.4448	66.7	31.2	1.0	1.0
299	1286.7	34.5636	62.7	31.1	0.2	6.0
300	1300.5	65.2131	73.8	20.5	0.9	4.9
301	1305.5	38.6462	72.6	19.7	0.6	7.0
302	1307.0	35.9490	74.0	19.2	0.7	6.1
303	1307.6	17.4866	76.0	19.7	0.9	3.4
304	1308.2	18.3913	76.8	19.0	1.1	3.0
305	1308.5	35.7582	75.0	20.2	0.8	4.0
306	1309.3	17.7758	76.2	19.5	0.7	3.6
307	1309.5	20.9819	76.3	19.5	0.7	3.6
308	1310.2	22.7767	77.1	19.1	0.8	3.0
309	1310.7	17.9154	78.4	18.2	0.8	2.6
310	1311.1	2.3740	77.9	18.3	0.8	2.9
311	1312.1	19.3174	78.1	18.4	0.9	2.6
312	1312.4	1.4279	74.7	21.8	0.9	2.7
313	1313.3	12.6758	78.9	18.0	0.8	2.4
314	1318.7	119.8308	68.4	21.8	0.2	9.6
315	1349.5	39.6816	74.2	21.4	0.8	3.6
316	1349.8	2.5093	79.1	17.9	0.3	2.6
317	1351.6	92.1079	64.4	23.9	0.5	11.2
318	1352.3	29.6145	65.3	25.0	0.9	8.7
319	1354.0	8.4179	68.7	23.6	0.8	6.9

Table S15: Mode contribution for polyalanine-15 in the β-strand configuration in percent for each vibrational mode n_{mode} and their corresponding frequencies in cm^{-1} and intensities in km/mol. As a subset we chose the modes 263–314. The modes 263–284 include the classical amide III vibrations, the modes 285–299 are the C^{α}-H(2) vibrations and the modes 300–314 are the C^{α}-H(1) vibrations.

n_{mode}	Frequency	Intensity	H	C	O	N
260	1131.5	31.4831	54.5	18.2	6.8	20.5
261	1136.9	7.0331	29.7	44.0	6.0	20.3
262	1138.0	15.5372	34.9	39.4	5.6	20.1
263	1140.2	20.5652	29.8	47.2	3.2	19.8
264	1140.6	45.5826	30.4	46.9	4.1	18.5
265	1150.8	16.7970	37.2	37.9	5.0	20.0
266	1153.5	41.8053	35.5	40.0	3.2	21.2
267	1153.8	15.2129	36.8	38.4	4.7	20.2
268	1161.8	359.4455	37.6	42.8	2.3	17.3
269	1162.1	16.3570	38.8	39.9	3.8	17.4
270	1180.3	74.0921	47.0	40.8	2.8	9.4
271	1183.1	233.3535	40.7	40.7	1.8	16.7
272	1185.7	64.6587	43.8	40.6	2.1	13.5
273	1192.9	56.0480	49.9	36.4	2.2	11.4
274	1194.3	129.3773	39.1	43.0	0.9	17.0
275	1194.8	23.7847	49.0	40.9	4.0	6.1
276	1198.8	128.4205	42.7	42.8	1.2	13.3
277	1199.8	122.1509	45.1	45.5	3.6	5.9
278	1200.9	30.1140	45.8	41.3	1.2	11.7
279	1203.3	48.6518	45.8	45.2	2.9	6.2
280	1205.1	19.0559	50.7	38.7	1.0	9.6
281	1207.9	68.6984	45.8	45.1	3.2	5.8
282	1209.9	15.5298	43.2	47.5	2.2	7.0
283	1213.4	37.6142	47.5	44.5	2.4	5.7
284	1218.6	16.3100	43.9	48.5	1.8	5.8
285	1253.6	1.0501	69.9	28.8	0.5	0.9
286	1258.1	7.0056	66.6	31.1	0.5	1.9
287	1260.9	13.7352	65.8	31.0	0.7	2.6
288	1262.2	3.6362	68.6	30.0	0.6	0.7
289	1263.4	6.6730	67.8	30.5	0.5	1.2
290	1263.7	1.7460	68.2	30.1	0.5	1.2

Table S16: Mode contribution for polyalanine-15 in the β-strand configuration in percent for each vibrational mode n_{mode} and their corresponding frequencies in cm^{-1} and intensities in km/mol (continued).

n_{mode}	Frequency	Intensity	H	C	O	N
291	1267.8	0.2057	68.4	29.9	0.9	0.8
292	1269.4	18.7006	69.0	28.8	0.5	1.8
293	1271.2	11.8708	67.7	30.0	0.4	1.9
294	1275.4	13.7173	66.4	31.1	1.2	1.3
295	1277.1	12.0790	68.0	30.8	0.4	0.7
296	1278.8	6.0927	68.2	29.6	0.4	1.8
297	1280.9	4.1585	64.8	33.8	0.4	1.0
298	1288.0	39.2943	71.8	24.5	0.4	3.3
299	1293.1	20.3033	76.1	20.4	0.1	3.4
300	1296.3	12.3098	67.3	28.1	0.6	4.1
301	1296.7	17.8298	59.9	37.8	1.7	0.7
302	1304.6	29.9908	75.2	21.4	1.0	2.4
303	1312.2	0.3968	78.6	17.1	1.9	2.3
304	1313.2	11.8122	80.7	15.8	1.1	2.4
305	1315.2	1.4334	76.8	18.9	1.3	3.1
306	1316.2	2.4932	78.0	18.0	1.6	2.5
307	1322.5	1.7241	73.6	22.8	1.4	2.2
308	1322.6	11.1612	66.2	30.7	2.0	1.1
309	1328.1	20.2955	71.3	25.3	1.7	1.7
310	1334.7	8.3473	70.1	25.9	1.8	2.1
311	1337.5	9.8166	72.1	24.2	2.0	1.7
312	1337.9	32.8608	69.5	26.5	1.5	2.5
313	1342.2	11.6338	70.6	26.0	1.9	1.4
314	1345.9	43.9782	78.3	21.4	0.1	0.2
315	1346.3	3.1134	74.3	22.9	1.5	1.2
316	1347.2	22.4050	79.7	19.7	0.3	0.3
317	1352.5	6.1204	81.3	18.5	0.1	0.2
318	1353.8	8.2366	80.6	19.0	0.1	0.2
319	1354.3	17.3792	80.1	19.6	0.1	0.2
320	1355.2	4.2381	79.6	20.1	0.1	0.2
321	1356.6	19.4220	80.2	19.4	0.1	0.3
322	1357.0	12.7719	80.0	19.8	0.1	0.1
323	1358.2	23.8740	79.0	20.6	0.1	0.3
324	1358.6	18.9989	80.6	19.3	0.1	0.1
325	1359.6	15.8913	79.7	20.0	0.1	0.2
326	1359.8	15.3701	79.3	20.4	0.1	0.2
327	1360.6	5.1115	77.8	21.7	0.2	0.4
328	1361.7	5.9110	78.6	21.0	0.1	0.3
329	1361.7	2.1375	79.8	19.9	0.2	0.2

Table S17: Mode contribution for polyalanine-16 in the β-hairpin configuration in percent for each vibrational mode $n_{\mathbf{mode}}$ and their corresponding frequencies in cm^{-1} and intensities in km/mol. As a subset we chose the modes 286–334. The modes 263–284 include the classical amide III vibrations, the modes 285–299 are the C^{α}-H(2) vibrations and the modes 300–314 are the C^{α}-H(1) vibrations.

n_{mode}	Frequency	Intensity	H	C	O	N
285	1162.8	150.7433	27.2	44.3	1.5	27.0
286	1173.1	19.4036	60.8	28.7	3.4	7.1
287	1175.4	58.8674	56.5	30.2	3.7	9.5
288	1187.8	4.5598	60.4	26.7	3.6	9.2
289	1189.3	41.4003	62.4	25.2	4.1	8.4
290	1196.5	120.4149	53.2	34.6	2.8	9.3
291	1199.5	87.9429	56.7	26.5	3.6	13.2
292	1204.6	67.8901	51.0	34.0	3.7	11.3
293	1206.6	21.2550	50.2	36.4	3.6	9.8
294	1207.5	52.6791	52.9	30.8	3.5	12.8
295	1207.9	80.3929	55.2	28.8	3.6	12.5
296	1209.4	6.7441	52.1	32.9	3.2	11.8
297	1213.2	10.9662	53.9	30.5	2.9	12.7
298	1214.7	16.5863	53.3	32.2	2.7	11.9
299	1217.3	19.6699	51.5	34.5	2.6	11.3
300	1217.6	30.0456	49.0	37.0	2.8	11.2
301	1220.6	69.2338	47.8	36.6	3.1	12.5
302	1239.4	33.3701	50.7	39.8	4.1	5.4
303	1255.4	21.6284	63.6	33.3	0.6	2.5
304	1255.9	2.1859	60.4	35.6	0.7	3.3
305	1259.8	30.1835	60.5	36.9	1.5	1.2
306	1262.1	9.7106	61.7	35.9	1.0	1.4
307	1267.7	23.3313	64.0	33.4	1.0	1.6
308	1272.0	36.9890	59.9	37.9	1.3	0.9
309	1275.2	2.0794	68.1	29.4	1.3	1.3
310	1276.2	30.9738	64.0	33.8	1.2	1.1
311	1280.2	13.8573	60.1	37.2	1.3	1.5
312	1280.4	1.5355	61.0	36.3	1.0	1.7
313	1281.2	1.6550	70.1	27.8	0.6	1.4
314	1283.8	5.6657	69.2	29.6	0.7	0.5
315	1285.3	2.7730	74.0	23.3	0.5	2.3

Table S18: Mode contribution for polyalanine-16 in the β-hairpin configuration in percent for each vibrational mode $n_{\mathbf{mode}}$ and their corresponding frequencies in cm^{-1} and intensities in km/mol (continued).

n_{mode}	Frequency	Intensity	H	C	O	N
316	1297.9	12.6961	64.9	32.1	1.6	1.4
317	1306.3	7.2533	70.3	26.7	1.8	1.3
318	1308.7	12.6005	74.7	22.9	1.3	1.1
319	1309.7	45.0330	80.5	16.0	1.1	2.5
320	1310.1	2.6634	74.0	22.6	1.1	2.3
321	1310.6	0.5467	75.1	22.6	0.8	1.4
322	1311.6	29.9112	76.8	20.4	0.6	2.1
323	1315.3	2.5272	77.7	19.4	1.0	1.8
324	1317.3	14.8964	76.5	21.3	0.5	1.7
325	1321.1	7.3151	78.3	19.5	0.5	1.8
326	1324.4	5.5179	79.4	18.1	0.1	2.4
327	1326.7	7.0089	64.3	31.1	2.6	2.1
328	1329.1	13.1058	63.0	32.3	2.6	2.0
329	1330.4	28.2457	69.3	27.4	1.3	1.9
330	1332.0	16.0944	67.3	29.4	1.4	1.9
331	1335.4	45.5741	64.5	31.5	1.9	2.1
332	1338.5	5.9397	71.7	25.5	1.2	1.6
333	1343.5	25.3555	72.6	23.8	1.9	1.7
334	1344.5	43.3402	65.9	30.0	2.4	1.8
335	1346.6	46.0624	76.3	23.3	0.1	0.3

References

(1) Hanwell, M. D.; Curtis, D. E.; Lonie, D. C.; Vandermeersch, T.; Zurek, E.; Hutchison, G. R. Avogadro: an advanced semantic chemical editor, visualization, and analysis platform. *J. Cheminformatics* **2012**, *4*, 17.

(2) Avogadro: an open-source molecular builder and visualization tool, Version 1.2.0. URL: https://avogadro.cc/ (accessed 2023-10-04).

(3) Kubelka, J.; Keiderling, T. A. Differentiation of β-Sheet-Forming Structures: Ab Initio-Based Simulations of IR Absorption and Vibrational CD for Model Peptide and Protein β-Sheets. *J. Am. Chem. Soc.* **2001**, *123*, 12048–12058.

(4) Humphrey, W.; Dalke, A.; Schulten, K. VMD — Visual Molecular Dynamics. *J. Mol. Graphics* **1996**, *14*, 33–38.

(5) Lindorff-Larsen, K.; Piana, S.; Palmo, K.; Maragakis, P.; Klepeis, J. L.; Dror, R. O.; Shaw, D. E. Improved side-chain torsion potentials for the Amber ff99SB protein force field. *Proteins: Struct., Funct., Bioinf.* **2010**, *78*, 1950–1958.

(6) Van Der Spoel, D.; Lindahl, E.; Hess, B.; Groenhof, G.; Mark, A. E.; Berendsen, H. J. C. GROMACS: Fast, flexible, and free. *J. Comput. Chem.* **2005**, *26*, 1701–1718.

(7) Jorgensen, W. L.; Chandrasekhar, J.; Madura, J. D.; Impey, R. W.; Klein, M. L. Comparison of simple potential functions for simulating liquid water. *J. Chem. Phys.* **1983**, *79*, 926–935.

(8) Brüggemann, J.; Chekmeneva, M.; Wolter, M.; Jacob, Ch. R. Data Set: Structural Dependence of Extended Amide III Vibrations in Two-Dimensional Infrared Spectra. 2023; DOI: 10.5281/zenodo.8406331 (accessed 2023-10-04).

(9) Jacob, Ch. R.; Reiher, M. Localizing normal modes in large molecules. *J. Chem. Phys.* **2009**, *130*, 084106.

(10) Panek, P. T.; Jacob, Ch. R. Anharmonic Theoretical Vibrational Spectroscopy of Polypeptides. *J. Phys. Chem. Lett.* **2016**, *7*, 3084–3090.

(11) Brüggemann, J.; Wolter, M.; Jacob, Ch. R. Quantum-chemical calculation of two-dimensional infrared spectra using localized-mode VSCF/VCI. *J. Chem. Phys.* **2022**, *157*, 244107.

(12) Panek, P. T.; Jacob, Ch. R. Efficient Calculation of Anharmonic Vibrational Spectra of Large Molecules with Localized Modes. *ChemPhysChem* **2014**, *15*, 3365–3377.

(13) Panek, P. T.; Jacob, Ch. R. On the benefits of localized modes in anharmonic vibrational calculations for small molecules. *J. Chem. Phys.* **2016**, *144*, 164111.

(14) Panek, P. T.; Hoeske, A. A.; Jacob, Ch. R. On the choice of coordinates in anharmonic theoretical vibrational spectroscopy: Harmonic vs. anharmonic coupling in vibrational configuration interaction. *J. Chem. Phys.* **2019**, *150*, 054107.

(15) Hamm, P.; Zanni, M. *Concepts and Methods of 2D Infrared Spectroscopy*, 1st ed.; Cambridge University Press: Cambridge, 2011.

(16) Mukamel, S. *Principles of Nonlinear Optical Spectroscopy*; Oxford University Press: New York, 1999.

(17) Jansen, T. l. C.; Saito, S.; Jeon, J.; Cho, M. Theory of coherent two-dimensional vibrational spectroscopy. *J. Chem. Phys.* **2019**, *150*, 100901.
